# Balancing national economic policy outcomes for sustainable development

**DOI:** 10.1038/s41467-022-32415-9

**Published:** 2022-08-26

**Authors:** Mohammed Basheer, Victor Nechifor, Alvaro Calzadilla, Claudia Ringler, David Hulme, Julien J. Harou

**Affiliations:** 1 Department of Mechanical, Aerospace and Civil Engineering, The University of Manchester, Manchester, UK; 2 Institute for Sustainable Resources, University College London, London, UK; 3 European Commission, Joint Research Centre (JRC), Seville, Spain; 4 International Food Policy Research Institute (IFPRI), Washington DC, USA; 5 Global Development Institute, The University of Manchester, Manchester, UK; 6 Department of Civil, Environmental and Geomatic Engineering, University College London, London, UK

**Keywords:** Carbon and energy, Economics, Climate change, Politics, Decision making

## Abstract

The 2030 Sustainable Development Goals (SDGs) aim at jointly improving economic, social, and environmental outcomes for human prosperity and planetary health. However, designing national economic policies that support advancement across multiple Sustainable Development Goals is hindered by the complexities of multi-sector economies and often conflicting policies. To address this, we introduce a national-scale design framework that can enable policymakers to sift through complex, non-linear, multi-sector policy spaces to identify efficient policy portfolios that balance economic, social, and environmental goals. The framework combines economy-wide sustainability simulation and artificial intelligence-driven multiobjective, multi-SDG policy search and machine learning. The framework can support multi-sector, multi-actor policy deliberation to screen efficient policy portfolios. We demonstrate the utility of the framework for a case study of Egypt by identifying policy portfolios that achieve efficient mixes of poverty and inequality reduction, economic growth, and climate change mitigation. The results show that integrated policy strategies can help achieve sustainable development while balancing adverse economic, social, and political impacts of reforms.

## Abstract (web summary)

Selecting economic policies to achieve sustainable development is challenging due to the many sectors involved and the trade-offs implied. Artificial intelligence combined with economy-wide computer simulations can help.

## Introduction

The world is facing severe pressures that put sustainable development at risk for most people. Population growth, growing inequalities, climate change, and emerging zoonotic diseases are setting back many recent human well-being achievements while rapidly worsening planetary health^1–4^. Several global initiatives have been put forward to tackle these challenges and pressures, including the Millennium Development Goals (MDGs), the Paris Agreement^5^, and the Sustainable Development Goals (SDGs)^6^.

The SDGs^7^, unlike the MDGs, apply to all countries and include 17 goals, 169 targets, and 231 unique indicators that cut across the social, economic, and environmental dimensions of sustainability^8^. Most countries around the world ratified the Paris Climate Agreement^5^, intending to keep the global average temperature well below 2 degrees Celsius above the preindustrial era. Meeting the SDGs while mitigating climate change requires a substantial reduction in fossil fuel use and a rapid shift to cleaner energy sources^9^. The SDGs are inherently interlinked^10^, and their implementation requires cross-sectoral actions from governments, civil society, non-governmental organizations, the scientific community, and businesses.

Globally, progress towards achieving the targets of the SDGs is off-track and has remained spatially uneven^11^. In 2020, some progress was recorded in children and youth education, fighting communicable diseases, and providing safe drinking water; nevertheless, food security, environmental sustainability, and inequality deteriorated^11^. The coronavirus (COVID-19) pandemic reduced greenhouse gas emissions and benefited the environment due to reduced human activity in the short term. However, COVID-19 worsened the performance of several other SDGs, resulting in a high death toll, collapsing health systems, economic recession, and growing inequalities, with 150 million people possibly pushed into extreme poverty and an additional 100 million food insecure people^11–13^.

The uniqueness of countries and regions must be recognized and considered when designing national policies for sustainable development^14,15^. For instance, the relationship between Gross Domestic Product (GDP), income and income inequalities, energy consumption, and carbon dioxide (CO_2_) emissions varies greatly across low-, middle-, and high-income countries^16–19^. Environmental degradation is influenced by many factors, including urbanization, fossil fuel consumption, trade, and the level of democracy^20^. Foreign direct investment in low-income countries has been shown to increase environmental degradation, while tourism has generated adverse environmental impacts in high- and middle-income countries^20^. These national and regional variations in the determinants of sustainability performance are due to each country’s unique economic, political, institutional, social, cultural, and environmental characteristics.

Governments are usually charged with crafting national development policies but face challenges in optimizing across sectors and often fail to adequately assess the impact of biophysical realities on economic growth and vice versa. Ideally, policymakers and stakeholders would develop economic policies that create synergies and manage trade-offs between different goals^21–24^. For instance, climate change mitigation and adaptation actions could have short-term trade-offs with other SDGs, such as poverty reduction and economic growth^25,26^, but long-term positive impacts on multiple SDGs^26,27^. Increased use of fertilizers in agricultural production would enhance food security in some countries but would also increase environmental pollution and associated health impacts^3,28^. The actions needed to achieve progress on the SDGs are further complicated by the multiple disciplines, sectors, actors, and levels that need to be involved in decision-making. Policy decisions that focus on individual SDGs might grow trade-offs with several other SDGs^27^. Such conflicts can be reduced, minimized, or avoided through comprehensive analysis and inclusive dialogue considering various combinations of policy instruments. Planning for a sustainable future requires a paradigm shift from sectorally-siloed consideration of the SDGs to a holistic approach that considers multiple SDGs and their trade-offs and synergies^29^.

This study introduces a framework for designing and screening efficient national economic policy portfolios that are aligned with the SDGs. The framework combines economy-wide sustainability performance simulation and artificial intelligence (AI)-driven machine learning and multiobjective, multi-SDG policy design to search for efficient national economic policy portfolios that maximize synergies and balance trade-offs between the SDG targets. The framework can enable co-production and screening of efficient policy portfolios through multi-actor, multi-sector deliberation to create social and political backing and commitment. We demonstrate the framework for Egypt by unraveling the trade-offs and synergies resulting from integrated economic strategies aimed at advancing multiple SDGs. Egypt is a middle-income country and ranks second in Africa in terms of GDP, CO_2_ emissions, and energy subsidies as a share of GDP^30,31^. Based on the national poverty line, the poverty rate in Egypt stood at 32.5% in 2017, and the overall Gini index of household income was around 0.31 in the same year^31^. We develop, calibrate, and use a dynamic economy-wide model of Egypt to simulate the country’s performance in achieving targets related to SDG1 (no poverty), SDG8 (decent work and economic growth), SDG10 (reduced inequalities), and SDG13 (climate action). The efficient economic policy portfolios revealed by the AI-driven search are based on 1.8 million 15-year (2021–2035) dynamic simulations of Egypt’s economy. We apply machine learning (Random Forest Regression Algorithm) to the outcomes of the 1.8 million simulations to understand the effectiveness of different policy instruments in influencing sustainability performance. The results show that economic policy portfolios that combine changes in existing poverty reduction programs, producer taxes/subsidies, sales taxes, and income taxes can reduce Egypt’s income inequalities and greenhouse gas emissions and balance distributional impacts while maintaining at least baseline GDP growth. Multi-sector, multi-actor co-production of policy portfolios based on the outcomes of policy search can help develop consensus across often competing economic, social, and environmental government mandates and support an improved balancing of sustainability and economic growth goals.

## Results

### Policy design and screening framework for sustainable development

**Fig. 1:**
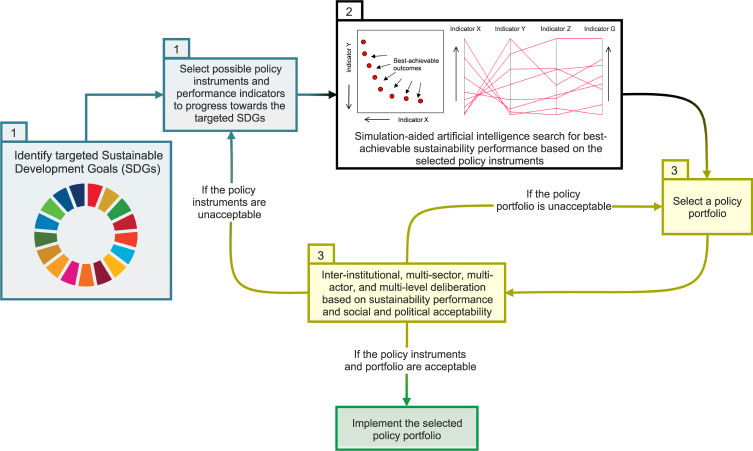
National economic policy design and screening framework for sustainable development. The framework includes three sub-processes: (1) identification of priorities aligned with the SDGs and associated policy instruments, (2) economy-wide simulation, artificial intelligence-driven multiobjective multi-SDG policy search, and machine learning of sustainability drivers, and (3) multi-sector, multi-actor policy screening and deliberation.

Figure 1 shows the national economic policy design and screening framework for sustainable development introduced in this study. The framework is based on an iterative process between three sub-processes: (1) identification of priorities aligned with the SDGs and associated policy instruments, (2) economy-wide simulation, AI-driven multiobjective, multi-SDG policy search, and machine learning of sustainability drivers, and (3) multi-sector, multi-actor policy portfolio screening and deliberation. In Fig. 1, the three sub-processes are numbered from 1 to 3.

Country-level progress towards sustainable development shows marked variations across the world^11,15^. Countries have different priorities, plans, and focus areas for the SDGs^32^. The first step in the proposed framework is, therefore, to identify sustainable development goals and targets and performance indicators based on national priorities. Furthermore, policy instruments that can be used to achieve the identified goals and targets are selected in this first step of the framework. We define “policy instrument” as an economic tool used to achieve sustainability goals. For example, poverty reduction can be achieved through direct government transfers to poor households; but it can also be accomplished indirectly by subsidizing the production activities on which poor households rely for their livelihoods.

Assessing the impacts of economic policies requires a holistic approach due to the forward and backward multiplier effects on different actors and sectors. Economy-wide models are the most commonly used tool to simulate national economic policies and their impacts on sustainability performance^33,34^. In the second step of the framework, an economy-wide model (i.e., computable general equilibrium model) is used to simulate the scope of different combinations of policy instruments regarding the social, economic, and environmental performance indicators identified in the first step. Economy-wide models can assess sustainability indicators that cut across multiple sectors and actors, even though they typically have been used for economic indicators; thus, such models can support inclusive policy deliberation and screening.

Scenario-based design of linear systems often use the superposition principle^35,36^, but economy-wide models are non-linear in their behavior^37^. For instance, doubling an economic shock does not lead to a doubled effect, and the impacts of policy instruments are not additive. Therefore, selecting and simulating a small set of policy scenarios to guide policy-making could be flawed by decision-making biases. Human decisions (including scenario selection) are often driven by rationality^38^. However, the perceptions and conceptions regarding interventions and their outcomes could influence this rationality, notably when decisions are related to financial choices or fatalities^38^. To overcome this issue, we couple the economy-wide model with an AI-driven multiobjective search to enable sifting through complex multi-SDG policy performance spaces to identify the most efficient policy portfolios based on combinations of the policy instruments identified in the first step. In this study, we define a “policy portfolio” as a parameterized set of policy instruments.

Crafting economic policy portfolios for sustainable development can yield thousands of policy options due to the complexity of multi-sector economies, the multidimensional nature of policy instruments, and the diversity of social, economic, and environmental sustainability targets. In the second step of the framework, the policy portfolios and their associated sustainability performances that resulted from the iterations of the multiobjective search algorithm are subjected to machine learning to understand the effectiveness of different policy instruments in influencing sustainability performance. Machine learning helps simplify the complexity of the interplay of policy instruments and multi-SDG performance, facilitating multi-sector multi-actor deliberation. The methods section provides a technical description of the second step of the framework.

The diverse policy options resulting from the second step of the framework can support inclusive multi-sector, multi-actor deliberation on policy reforms. While we have not yet conducted such a deliberation process in the case study application shown later in this paper, stakeholder deliberation based on artificial intelligence search and machine learning has been successfully applied in other disciplines^39–41^, and therefore we suggest it as a step in our proposed framework. Multiobjective search results can be useful for policy-making because they are transparent, policy-relevant, and provide options and not recommendations^42,43^. In the third step of the framework, stakeholders and actors representing different institutions, sectors, disciplines, and regions negotiate and screen the efficient policy portfolio options (generated in step 2). Such deliberation should aim for a balanced (i.e., socially acceptable) sustainability performance across time (e.g., temporal distribution of impacts), space (e.g., rural and urban regions), and income groups (e.g., wealthy and poor households); given the large set of policy portfolio options, deliberation would allow policymakers with different sectoral mandates to find common ground. Citizen assemblies, to advise on policy choices, are a mechanism that is increasingly being used as part of such deliberation processes^44^. Public-private partnerships can further support the implementation of associated measures^45^. Although multiobjective economic policies are complex, machine learning and interactive data visualization techniques can help stakeholders understand, explore, screen, and select policy portfolios and/or identify unacceptable options. Subsequently, a new or modified set of policy instruments for achieving the targeted SDGs could be explored. The new or revised policy instruments would then be used to generate a revised set of efficient policy portfolios to be integrated into the multi-sector multi-actor deliberation and co-production of sustainability reforms. At their most ambitious, such consultation mechanisms can seek to identify compromise portfolio options. Less ambitiously, they can help screen out socially unacceptable options.

### Balanced national economic policies in Africa improve sustainability

The African continent is currently far from achieving most of the SDGs by 2030 and has the lowest performance globally in many goals^11,46^. Sustainable development in Africa is challenged by poor governance, limited financial resources, high population growth rates, and the COVID-19 pandemic^46–48^. Africa’s contribution to global greenhouse gas emissions is low, at around 4% in 2019^49^, but the rate of growth of the continent’s emissions is increasing rapidly^50^. Without urgent decarbonization policies, Africa could lock in sizable greenhouse gas emissions for several decades in the future^51^ or end up with stranded assets^52^.

We apply the SDG economic policy design and screening framework to Egypt, a middle-income country in northern Africa. Egypt is the second-highest CO_2_ emitter on the continent (Supplementary Fig. 1) and faces economic and sustainability challenges. In 2016, Egypt contributed around 17% of Africa’s total CO_2_ emissions^31^, following a national increase of 55% from 2006 to 2016^49^. Furthermore, energy commodities are heavily subsidized^53^, with the level of energy subsidies ranking second in Africa as a share of GDP^30^. Although energy subsidies contribute to stabilizing the prices of energy-dependent commodities and increasing the output of some industries, they fuel CO_2_ emissions and fiscal deficits, can slow economic growth and diversification of energy portfolios, and grow inequalities across social groups^54^. In 2017, the overall Gini Index of Egypt was estimated at 0.31 by the World Bank^31^, indicating considerable income discrepancies.

We use the framework to identify efficient policy portfolios for Egypt’s economy aligned with targets related to five SDGs. These targets are enhancing GDP growth (SDG8), increasing rural and urban incomes (SDG1), reducing rural, urban, and overall income inequalities (SDG10), and lowering CO_2_ emissions (SDG13). These five SDG targets were selected because they are directly related to one of Egypt’s most pressing economic challenges: how to reduce commodity subsidies while lowering inequality and poverty and ensuring economic growth and environmental sustainability^55–57^. We developed, calibrated, and used a dynamic Computable General Equilibrium (CGE) model of Egypt’s economy to simulate the country’s performance in achieving these targets as well as the associated trade-offs and synergies. The economy-wide model was set up for the 2021–2035 period and was connected to a multiobjective evolutionary algorithm to search for efficient economic policy portfolios based on four incremental policy strategies. Following that, a machine learning approach was used to understand the drivers of sustainability performance. The multiobjective multi-SDG policy search process involved 1.8 million 15-year (2021–2035) dynamic simulations, from which a total of around 20 thousand efficient policy portfolios were identified. The four incremental integrated policy strategies used in designing sustainability policy portfolios for Egypt are: (I) Distribution and total amount of direct government transfers to households, (II) Distribution and total amount of direct government transfers to households and income taxes on households, (III) Producer taxes/subsidies on economic activities, distribution and total amount of direct government transfers to households, and income taxes on households, and (IV) Producer taxes/subsidies on economic activities, sales taxes/subsidies on commodities, distribution and the total amount of direct government transfers to households, and income taxes on households. The economy-wide model of Egypt was set up such that economic reforms are implemented gradually over five years from 2021 to 2025. For example, a tax increase of 5% is applied by adding a 1% increase annually over the 5-year assumed reform period. This reform period was selected to demonstrate the use of the framework and is customizable based on stakeholder preferences. Further details on the mathematical formulation of the economy-wide model and the multiobjective search can be found in the methods section.

**Fig. 2:**
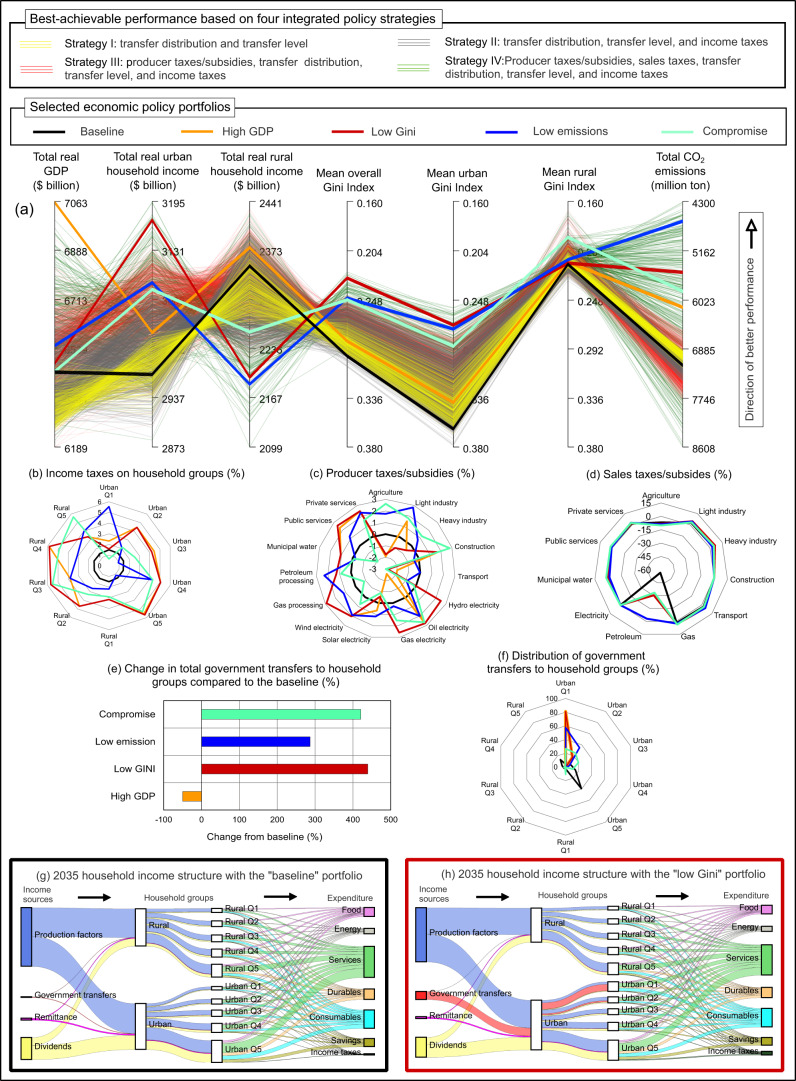
Sustainability performance of the Egyptian economy in 2021–2035. **a** parallel coordinates plot of the best-achievable aggregate performance based on four integrated policy strategies, **b**–**f** details of the economic policy portfolios associated with the five thick lines highlighted in panel (**a**), **g** Sankey diagram of the structure of household income and expenditure in 2035 with the baseline economic portfolio, and **h** Sankey diagram of the structure of household incomes and expenditures in 2035 in the low Gini economic portfolio. The thin lines in panel a represent all efficient portfolio options, while the thick lines highlight selected policy portfolios. The upward direction on each axis in panel (**a**) is desirable (i.e., a perfect policy would be a straight line across the top), and diagonal lines between axes indicate trade-offs. Supplementary Fig. 7 depicts a version of panel a with the efficient policy portfolios shown separately for each of the four integrated policy strategies. The number of economic policy portfolios (or lines) in panel (**a**) is 19723. The line and bar colors in panels (**b)** and (**c**) correspond to the thick lines with similar colors in panel (**a**) (i.e., selected economic policy portfolios). The Sankey diagrams in panels (**g**) and (**h**) show household income by source (left-most axis), recipient household group (two central axes), and expenditure (right-most axis). The boxes drawn around panels (**g**) and (**h**) correspond to the thick lines with similar colors in panel (**a**). The total gross domestic product (GDP) and household income values in panel (**a**) are discounted at 3%. CO_2_ stands for carbon dioxide; Q1–Q5 are household classes based on income quintiles from poorest to richest.

Figure 2a shows a parallel coordinates plot^58^ of Egypt’s sustainability performance from 2021 to 2035 under efficient economic policy portfolios generated in the second step of the SDG policy design and screening framework. The policy portfolios are based on four integrated policy strategies described above. For the purpose of this paper and in order to demonstrate the framework, the selected policy portfolios (thick lines in Fig. 2a except for the baseline) are assumed to result from multi-sector multi-actor negotiation and deliberation. Stakeholders from different backgrounds would target specific sustainability dimensions (e.g., high GDP, low Gini Index, or low emissions), but an efficient compromise policy portfolio could eventually be agreed upon based on deliberation and co-production of policies.

Figure 2a shows that the four examined integrated policy strategies have variable impacts on sustainability performance. Using policy strategy I (thin yellow lines) reduces income inequalities compared to the baseline (thick black line); however, this strategy slows economic growth because more government income is spent on households rather than on investment. Policy strategy II (thin grey lines) further reduces income inequalities and also improves economic growth compared to the baseline. Using policy strategy III (thin red lines) yields solutions that increase total GDP, increase urban and rural total incomes, and reduce total CO_2_ emissions compared to the baseline and strategy II. Finally, strategy IV (thin green lines) increases the sustainability performance space, leading to the lowest trade-offs between the targets compared to the three other strategies.

The selected sustainability policy portfolios (thick lines in Fig. 2a) show trade-offs between sustainability targets. For example, aiming to achieve high GDP results in only a slight reduction in income inequalities, whereas targeting low Gini indices results in a total GDP value close to the baseline. There is also a trade-off between rural and urban income (i.e., diagonal lines between the two axes). Sustainability performance under the low emissions portfolio shows a reduction in overall and urban inequalities, a reduction in rural income, an increase in urban income, and a slight improvement in GDP performance compared to the baseline. Overall inequalities decline because the increase in urban income mostly goes to poor urban households, which reduces the overall income gap between poor and rich households. Figure 2b–f and Supplementary Table 1 provide the numeric details of the five policy portfolios (including the baseline) shown as thick lines in Fig. 2a. Achieving high GDP growth involves a 50% reduction in the total amount of government transfers to households (Fig. 2e), increases of more than 100% in income taxes on households (Fig. 2b), a 50% reduction in subsidies on petroleum commodities (Fig. 2d), an increase in producer and sales subsidies on agriculture, new producer subsidies on manufacturing, and an increase in sales taxes on manufacturing commodities. In contrast, reducing income inequalities (i.e., low Gini portfolio) requires increasing government transfers to households by 440%, channeling most government transfers to poor urban households, and increasing income taxes on rich households (see Fig. 2b, c). Also, achieving low Gini requires introducing taxes on hydropower, oil, and gas electricity activities and subsidizing solar electricity producers. The low emissions portfolio involves reducing subsidies on petroleum commodities by around 90%, coupled with a 286% increase in the total amount of government transfers to households to mitigate the rise in commodity prices resulting from the reduction in fossil fuel subsidies. As Fig. 2a shows, the compromise policy portfolio reduces CO_2_ emissions, reduces rural, urban, and overall income inequalities, and achieves economic growth similar to the baseline portfolio. It is worth noting that the compromise policy portfolio does not result from averaging the other portfolios, as the economy behaves non-linearly, highlighting the importance of the multiobjective search within the policy design and screening framework.

**Fig. 3:**
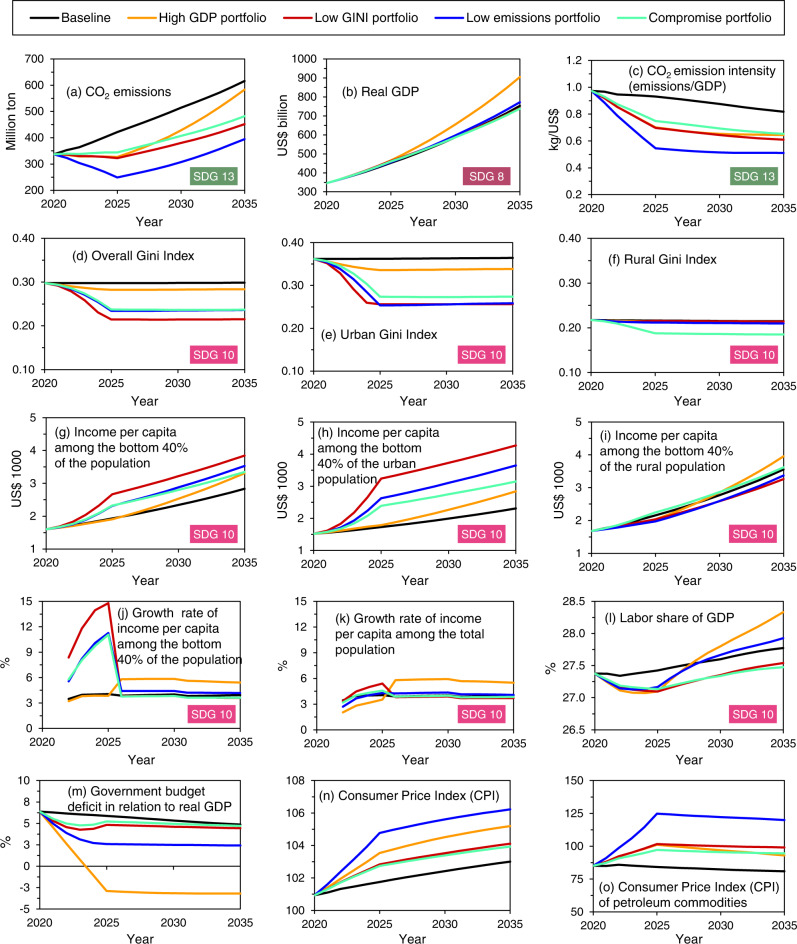
Temporal evolution of Egyptian sustainability performance indicators. **a**–**o** time series of sustainability indicators based on five economic policy portfolios. The time series depicted in this figure correspond to the thick lines with similar colors in Fig. 2a. Details on the economic policy portfolios associated with the time series are provided in Fig. 2b–f and Supplementary Table 1. GDP stands for gross domestic product and CO_2_ stands for carbon dioxide.

Figure 3 depicts time series of sustainability performance indicators for the five economic policy portfolios highlighted as thick lines in Fig. 2a. With the high GDP policy portfolio, CO_2_ emissions (Fig. 3a) decline over the 5-year assumed reform period but increase steadily afterward due to a rapid increase in the economy’s energy needs to enhance economic growth (Fig. 3b). However, the budget deficit (Fig. 3m) declines most under the high GDP policy portfolio due to reductions in government transfers to households and subsidies on petroleum products. The high GDP policy portfolio shows the highest increase in the labor share of GDP (i.e., labor income divided by real GDP; see Fig. 3l) and the highest overall growth in income per capita (Fig. 3k). The low Gini policy portfolio yields the highest decrease in income inequalities (Fig. 3d–f), the highest increase in the income of the poorest 40% of the total and urban Egyptian populations (Fig. 3g–i), a decrease in CO_2_ emissions and emission intensities (Fig. 3a, c), and approximately similar economic growth to the baseline portfolio (Fig. 3b). The low emissions policy portfolio leads to the lowest CO_2_ emission intensity (Fig. 3c) and increases the income of the poorest 40% of the population (Fig. 3g– i). The low emissions economic policy portfolio results in the highest increase in the overall consumer price index (Fig. 3n) and the price index of petroleum commodities (Fig. 3o). The rise in commodity prices is a major challenge associated with subsidy reforms. Reductions in energy subsidies reduce welfare because households and industries face higher energy prices and an increase in the prices of other commodities that use energy as an intermediate input^59^.

**Fig. 4:**
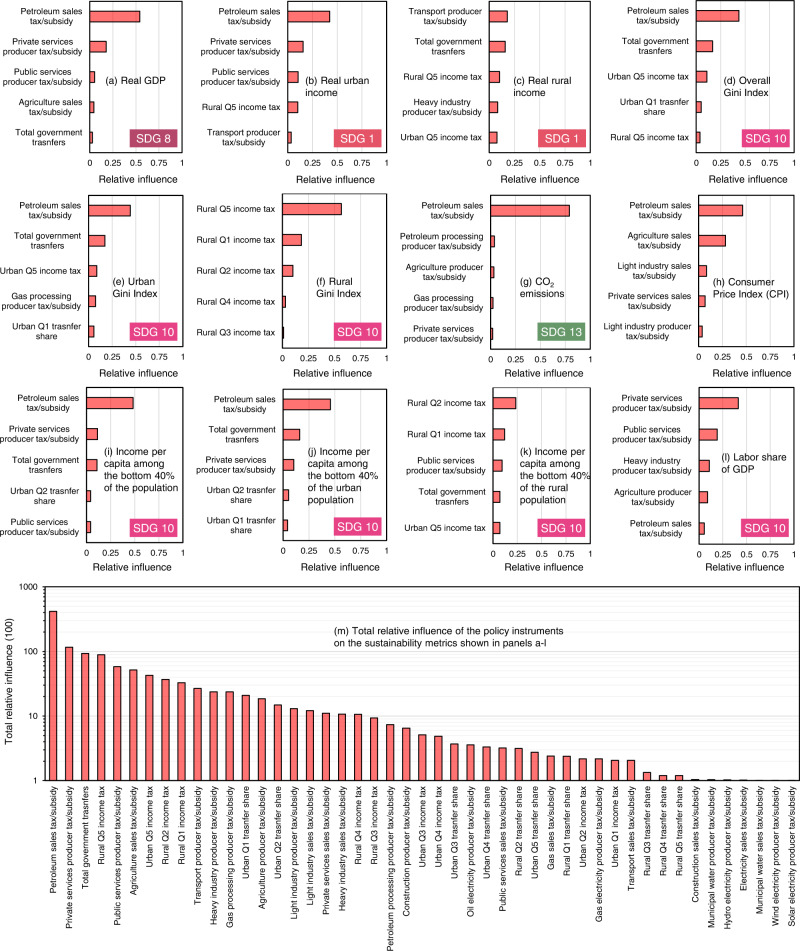
Rankings of policy instruments based on their relative influence on twelve Egyptian sustainability performance indicators. **a**–**l** the five most influential instruments for each sustainability performance indicator. **m** the overall relative influence of each policy instrument on the twelve sustainability performance indicators. The relative influence values shown on the *x*-axes of panels (**a**–**l**) vary from zero to one. A zero value means the policy instrument does not influence the performance indicator, whereas one indicates that the policy instrument is the only influencer of the performance indicator. For each performance indicator, the sum of the relative influence values for all instruments is one. Panel (**m**) shows the sum of the relative influence values for each of the twelve policy instruments multiplied by a hundred and plotted on a logarithmic scale. Therefore, the values in panel (**m**) can range from zero to twelve hundred, with zero indicating that the instrument does not influence any of the twelve performance indicators, and twelve hundred meaning that the policy instrument is the only influencer of all twelve performance indicators. GDP stands for gross domestic product, CO_2_ stands for carbon dioxide, and Q1 to Q5 are household classes based on income quintiles from poorest to richest.

Policy instruments vary in their effectiveness in impacting the SDGs. In this study, we used a machine learning method to assess the effectiveness of policy instruments in influencing sustainability targets (Fig. 4). As Fig. 4a–l show, some policy instruments are effective for specific targets, whereas others affect multiple targets. Overall, changing the tax/subsidy on petroleum sales is the most effective policy instrument for influencing multiple sustainability targets, followed by a producer tax/subsidy on private services and then government transfers to households (Fig. 4m). The degree of influence of policy instruments also depends on the range within which they are allowed to vary. Supplementary Table 2 reports the upper and lower bounds assumed for each policy instrument.

## Discussion

Realizing the SDGs necessitates a profound change in decision-making by adopting interdisciplinarity and multi-sector, multi-actor co-production of policies^60–62^. Achieving sustainable development involves trade-offs and synergies between different goals and targets^3,26,63^. Therefore, policies designed to achieve individual SDG targets are no longer viable, as such an approach reduces policy and sectoral synergies^29^. A system-wide perspective helps better understand the trade-offs and synergies of attaining the SDGs^64^. Reactivating national, cross-sector planning systems, as illustrated by Vietnam’s successful progress towards the MDGs, SDGs, and minimizing Covid-19 impacts, should be encouraged^32^. This paper introduces a national-scale economic policy design and screening framework for sustainable development. The proposed framework offers an approach that could help reduce the gaps between science and policy, public and private sectors, governments and civil societies, and public institutions through inclusive dialogue on economic policy design for sustainable development. Because the political dimension of simulating sustainable development is important^65^, the sustainability policy design and screening framework would enable politicians to better weigh SDG reforms against election promises and other political goals informed by technical analysis and the views and goals of various stakeholders. Citizen assemblies or other types of multistakeholder platforms convened by the government or civil society could provide a mechanism for fostering such stakeholder engagement^66,67^.

Simulation models have uncertainties that can be characterized and quantified using, for example, sensitivity analysis^68^. For our case study, we conducted sensitivity analyses around key model parameters (i.e., oil price, total factor productivity, production and international trade elasticities, foreign savings, and labor supply) to understand how sensitive the optimization objectives and policy choices are to model parameterization. Supplementary Fig. 2 and Supplementary Table 3 show the results of the sensitivity analyses under all the efficient policy portfolios presented in Fig. 2a. These analyses show that while the objective values have varying degrees of sensitivity to some model parameters, policy choices remain robust to the uncertainty of the tested parameters because changes to the model parameters examined do not result in large structural changes, given the short simulation period considered (15 years).

Egyptian inter-ministerial entities, such as the committee formed to follow national SDG progress, can benefit from the proposed framework in evaluating economic interventions that affect multiple sectors. Although Egypt has had success in progressing toward some SDGs, as shown in the country’s 2021 Voluntary National Review, further interventions are needed to meet the goals by 2030, notably after the impact of the COVID-19 pandemic^57^. Recent data show that the Greater Cairo urban periphery made progress in infrastructure and education sustainability but deteriorated in economic and environmental targets^69^. A shift from consumption expenditures, notably subsidies, to investment expenditures can contribute to Egypt’s progress towards sustainable development, but such an economic shift should be complemented with policies to support goals that are loosely related to economic growth^70^. Developing a roadmap for implementing the SDGs at the governorate level in Egypt is key to ensuring rapid, inclusive, and balanced sustainable development^71^.

Economic policy reforms aimed to achieve sustainable development and increased equity could produce detrimental short-term social and political consequences. Such policies are like “painful surgeries,” but the sooner they are done, the better. For instance, energy subsidy reforms can increase commodity prices, hit the poor the hardest, and lead to popular discontent and protest, fueling political instabilities^72^. This would make the sustainability transformation politically and socially unappealing. The proposed framework can help identify efficient policy portfolios that balance the distributional impacts of policy reforms, highlight political risks by pointing out winners and losers, and thus help strengthen political and social support and buy-in for sustainability. Furthermore, sustainable economic reforms could qualify Heavily Indebted Poor Countries (HIPC) for external debt relief by the World Bank, the International Monitory Fund, and other multilateral entities^73^, potentially attracting low-interest external financing for sustainable development investments.

The framework introduced in this study requires a joined-up strategic approach to sustainability decision-making with a partnership spanning government, civil society, non-governmental organizations, the scientific community, and the private sector. Such an alliance combined with multi-SDG policy design can help countries navigate towards a more sustainable future.

## Methods

### General description of the second step of the framework

The second step of the sustainability policy design and screening framework introduced in this study involves coupling a national-scale economy-wide model with AI-driven multiobjective search and machine learning algorithms to identify efficient (approximately Pareto-optimal) policy portfolios based on combinations of different policy instruments. This approach enables navigating complex, multidimensional, non-linear sustainability performance spaces to search for efficient economic policy portfolio options that maximize synergies and balance trade-offs between sustainable development targets. Below we describe the economy-wide model, followed by a description of the linkages with multiobjective search and machine learning processes.

### Economy-wide sustainability simulation

We use a dynamic-recursive CGE model to simulate sustainability performance and the impacts of different national economic policies. A CGE model enables assessing the economy-wide impacts of alternative policy options on key metrics, such as income and expenditures of different economic actors (e.g., government, household groups, industries, enterprises, and the rest of the world), commodity production, consumption, trade, and prices, factor endowments and prices, and CO_2_ emissions. The CGE model used in this study is based on the standard open-source CGE model of the International Food Policy Research Institute (IFPRI)^74^. IFPRI’s CGE model is a single-country, open-economy model and follows the well-known “small open-economy” assumption used with small economies, whereby the economy engages in international trade but does not influence world prices. The reader is referred to Lofgren et al. (2002)^74^ for the details of the mathematical formulation of IFPRI’s standard CGE model. IFPRI’s original model was customized as follows. Commodities are produced by economic activities using a nested three-level process. Supplementary Fig. 3 shows production configuration in the CGE model. At the top level of the production process, a bundle of value-added-energy is combined with composite intermediate inputs using the Leontief Function^75^, maintaining fixed ratios of these two inputs. At the middle level of the production process, value-added and energy are combined to form the value-added-energy bundle using Constant Elasticity of Substitution (CES) functions^76^, such that input quantities of energy and value-added are based on their relative prices but constrained by substitution elasticities. At the bottom level of the production nesting, CES functions are used to combine labor, land, and different capital types into value-added. Similarly, electricity and other types of energy are incorporated into an energy bundle using CES functions. Supplementary Fig. 4 depicts the specification of household consumption in the CGE model. A two-level demand system is used to simulate household commodity consumption. At the top level, household consumption budget is divided across five commodity categories through a Linear Expenditure System (LES)^77^ specification separating subsistence from supernumerary consumption. At the second level, households can substitute between commodities within each commodity category based on CES functions subject to substitution elasticities.

The economy-wide model of Egypt was set up to run over the 2021–2035 period. The model includes four economic agents: the government, households, enterprises, and the rest of the world. Households are disaggregated into ten groups based on regional affiliation (i.e., rural and urban) and level of wealth (five income quintiles). Fifteen production activities are included in Egypt’s CGE model: agriculture, light industry, heavy industry, construction, transport, hydroelectricity, oil electricity, gas electricity, solar electricity, wind electricity, gas processing, petroleum processing, municipal water, public services, and private services. These activities produce 11 different commodities including electricity, which is generated by four different technologies. The selected sectoral aggregation comprehensively represents the Egyptian economy while focusing on the key sectors needed to address the five SDGs considered. Producers use three types of productive factors: labor, land, and capital. Capital is then specified as sector-specific for the electricity and water economic activities. The baseline scenario was calibrated to follow economic performance under the Shared Socioeconomic Pathway “middle of the road” scenario (SSP2)^78–80^. Accordingly, labor growth (16-64 age group), population growth, urbanization, and total factor productivity are updated exogenously following SSP2 assumptions. CO_2_ emissions are calculated in the CGE model based on the national consumption of petroleum, gas, and coal by households, production activities, firms, and the government. Specific CO_2_ emission factors (tons of carbon/Tera Joule) were used for petroleum, gas, and coal to calculate emissions based on the national consumption of these three commodities.

The price of petroleum on the international market was set up to follow the World Bank projection for the average price of crude oil^81^. Prices of other commodities on the international market are fixed, assuming a small open economy setup^82^. The balance of payments is determined by exogenous foreign savings and variable exchange rate. Savings are based on fixed saving propensities of different economic agents. Annual savings are used dynamically to invest in various capital types except for hydro capital. This is because no major future hydropower investments are expected in Egypt^83^. Investment is allocated between different capital types based on their relative rates of return. Investment premiums and investment relocation are applied exogenously to electricity capital types to ensure the growth of baseline CO_2_ emissions follows the regional projection of the Middle East and North Africa under SSP2-4.5^79,84^. Egypt’s CGE model was calibrated to a 2011 Social Accounting Matrix (SAM) produced by IFPRI^85^. In addition, we used the GTAP-Power 10 database for the year 2014 from the Global Trade Analysis Project^86^ to disaggregate the electricity sectors of the SAM. Then the model was run dynamically over 2011-2020 to bring the status of the economy closer to the present before proceeding with the 2021–2035 simulation period of our case study analysis.

### Economy-wide simulation coupled with artificial intelligence processes

We linked the economy-wide dynamic model to an AI-driven multiobjective evolutionary algorithm (MOEA) to design efficient (or approximately Pareto-optimal) national economic policy portfolios. Supplementary Fig. 5 depicts a flowchart of the interaction between the CGE model and the evolutionary algorithm. The process starts with generating an initial policy portfolio based on a specified set of policy instruments (yellow box in Supplementary Fig. 5). Next, the policy portfolio is applied to the CGE model, which runs dynamically over a multi-year simulation timeframe to compute performance indicators used as objectives that are maximized or minimized in the search process. The values of the performance indicators and the policy portfolio are then stored. A stopping criterion is checked before proceeding to the next iteration. In this study, a minimum number of iterations (90 thousand in the case study application) is used as a stopping criterion. If the stopping criterion is not met, the search process proceeds to the next iteration, in which the search algorithm generates a new policy portfolio. The evolution of multiobjective performance in the previous iterations informs the generation of new policy portfolios. Once the stopping criterion is met, a non-dominated sorting is performed for all stored solutions to filter efficient (or approximately Pareto-optimal) policy designs.

Multiobjective evolutionary algorithms search for an approximately optimal set of solutions by imitating the natural biological evolution process^87,88^. This involves three main steps: selection, crossover, and mutation. The selection step resembles “survival of the fittest,” whereby the best performing set of solutions (from a generation of iterations between the simulation model and the MOEA) is selected based on their performance in achieving the optimization objectives (i.e., domination). The selected solutions are then used to imitate natural reproduction by producing new solutions (or children) based on a crossover of the characteristics of the original solutions (or parents). Random mutations are then introduced to the children to ensure that the new solutions not only improve as a result of parents’ crossover but also due to new random features. The children then enter into the selection process and are treated like new parents. The process of selection, crossover, and mutation continues until a stopping criterion is met, as explained earlier and in Supplementary Fig. 5.

In the case study application, we specified seven optimization objectives for the search algorithm: maximizing total discounted real GDP (Supplementary Equation 1), maximizing total discounted net real urban household income (Supplementary Equation 2), maximizing total discounted net real rural household income (Supplementary Equation 3), minimizing the mean overall Gini Index (Supplementary Equation 4), minimizing the mean urban Gini Index (Supplementary Equation 5), minimizing the mean rural Gini Index (Supplementary Equation 6), and minimizing total CO_2_ emissions (Supplementary Equation 7).

Four configurations of decision variables (i.e., four integrated policy strategies; see Fig. 2) were used, and the multiobjective search process was performed for each of these configurations separately. The upper and lower limits of each decision variable (i.e., each parameter in the policy instruments) used in the multiobjective search process are provided in Supplementary Table 2. The search process was performed with five random seeds (i.e., five random starting conditions for the search algorithm) for each of the four integrated policy strategies. A minimum of 90 thousand iterations (i.e., function evaluations) was specified as a stopping criterion for each seed (i.e., a total of 1.8 million simulations resulting from five seeds multiplied by four policy strategies multiplied by 90 thousand iterations). Supplementary Fig. 6 shows the hypervolume^89^ with each of the five random seeds for each of the four integrated policy strategies. Generally, the figure shows that the hypervolume stabilizes before the 90 thousand iteration limit, indicating convergence to approximately Pareto-optimal policy portfolios. We used the Non-dominated Sorting Genetic Algorithm III (NSGA-III)^88^ to search for efficient economic policy portfolios. NSGA-III uses reference points to guide the search and to find well-converged diverse solutions for problems with up to 15 objectives^88^.

We used the Random Forest Regression Machine Learning Algorithm^90^ to determine the relative influence of policy instruments on sustainability targets. A Random Forest Regression model is an ensemble of regression trees created based on random sampling from the training data and random selection of input features^91^. Random forest regression models make predictions by aggregating the predictions of the individual tree ensemble members based on the majority vote (i.e., the mean prediction)^91^. The relative importance of different input features to the Random Forest model was quantified by calculating the importance of features (i.e., decision variables) in predicting the targets (i.e., optimization objectives). In the case study application, the 1.8 million outcomes of the multiobjective search process were randomly split into training (80% of the data) and testing (20% of the data) datasets. The training dataset was used to train ensembles of 100 tree predictors, and the testing dataset was used to assess the performance of the machine learning models. Twelve machine learning models were developed, one for each of the performance indicator shown in Fig. 4a–l. Sensitivity analysis was performed around the maximum tree depth of each of the models. Tree depths ranging from 1 to 50 were tested for each of the 12 machine learning models. Then the lowest tree depth that does not result in over-fitting or under-fitting the testing or training data was selected for each model. Supplementary Fig. 8 shows the performance of the machine learning models with the testing and training data and the selected maximum tree depth for each of the twelve models.

The Platypus Python optimization library^92^, which includes NSGA-III, was used in performing the multiobjective search. The Scikit-learn Python library^93^ was used to perform machine learning of sustainability drivers based on the Random Forest Regression Algorithm. The Python Network Simulation framework (Pynsim)^94^ was used to couple the CGE model with Platypus through the General Algebraic Modeling System (GAMS)^95^ Python Application Programming Interface (API).

### Reporting summary

Further information on research design is available in the Nature Research Reporting Summary linked to this article.

## Supplementary information

Supplementary Information

Reporting Summary

## Data Availability

The data that support the findings of this study been deposited in a Zenodo repository under the accession code: 10.5281/zenodo.6533977. The baseline population, labor, urbanization, and economic growth data of Egypt associated with the SSP-2 can be accessed from the International Institute for Applied System Analysis (IIASA) database: https://tntcat.iiasa.ac.at/SspDb/dsd?Action=htmlpage&page=10. Egypt’s 2011 SAM can accessed from: http://ebrary.ifpri.org/cdm/ref/collection/p15738coll2/id/130736. Crude oil price projections can be retrieved from: https://knoema.com/infographics/yxptpab/crude-oil-price-forecast-2021-2022-and-long-term-to-2050. The GTAP-Power 10 database can be obtained from: https://www.gtap.agecon.purdue.edu/resources/res_display.asp?RecordID=5938.

## Code availability

The standard CGE model of the International Food Policy Research Institute (IFPRI) is open-source and freely accessible through the following link: https://www.ifpri.org/publication/standard-computable-general-equilibrium-cge-model-gams-0. The Python Network Simulation framework (Pynsim) is open-source and freely available in the following repository: https://github.com/UMWRG/pynsim. The multiobjective Non-dominated Sorting Genetic Algorithm (NSGA-III) used in the SDG economic policy design and screening framework is open-source and freely available in the following repository: https://github.com/Project-Platypus/Platypus. The Random Forest Regression, a machine learning algorithm used for understanding the drivers of sustainability performance, is open-source and freely available at: https://github.com/scikit-learn/scikit-learn. The General Algebraic Modeling System (GAMS) and the GAMS Python API can be obtained from: https://www.gams.com/.

## References

[1] United Nations. *World population prospects 2019: Highlights*. *United Nations, Department of Economic and Social Affairs, Population Division* https://population.un.org/wpp/Publications/Files/WPP2019_Highlights.pdf (2019).

[2] Steffen, W. et al. Planetary boundaries: guiding human development on a changing planet. *Science* **347**, 6223 (2015). 10.1126/science.1259855 25592418

[3] Lu Y, Nakicenovic N, Visbeck M, Stevance A-S (2015). Five priorities for the UN sustainable development goals. Nature.

[4] World Health Organization. Climate change and health. (2008).

[5] United Nations Framework Convention on Climate Change. *Paris Agreement*. (2015).

[6] United Nations. The future we want: Outcome document of the United Nations Conference on Sustainable Development. *Rio* + *20 United Nations Conference on Sustainable Development* (2012).

[7] Glaser G (2012). Base sustainable development goals on science. Nature.

[8] United Nations General Assembly. Transforming our world: the 2030 Agenda for Sustainable Development. *United Nations* (2015).

[9] Panwar NL, Kaushik SC, Kothari S (2011). Role of renewable energy sources in environmental protection: a review. Renew. Sustain. Energy Rev..

[10] Le Blanc D (2015). Towards integration at last? the sustainable development goals as a network of targets. Sustain. Dev..

[11] United Nations. *The Sustainable Development Goals report 2020* https://unstats.un.org/sdgs/report/2020/The-Sustainable-Development-Goals-Report-2020.pdf (2020).

[12] FAO, IFAD, UNICEF & WEP. The state of food security and nutrition in the world 2021.Transforming food systems for food security, improved nutrition and affordable healthy diets for all. 10.4060/ca9692en (2021).

[13] The World Bank. COVID-19 to add as many as 150 million extreme poor by 2021. https://www.worldbank.org/en/news/press-release/2020/10/07/covid-19-to-add-as-many-as-150-million-extreme-poor-by-2021 (2020).

[14] United Nations General Assembly. Resolution adopted by the General Assembly on 27 July 2012. *United Nations* (2012).

[15] Zheng X (2021). Consideration of culture is vital if we are to achieve the Sustainable Development Goals. One Earth.

[16] Al Mamun M, Sohag K, Hannan Mia MA, Salah Uddin G, Ozturk I (2014). Regional differences in the dynamic linkage between CO_2_ emissions, sectoral output and economic growth. Renew. Sustain. Energy Rev..

[17] Antonakakis N, Chatziantoniou I, Filis G (2017). Energy consumption, CO2 emissions, and economic growth: an ethical dilemma. Renew. Sustain. Energy Rev..

[18] Narayan PK, Saboori B, Soleymani A (2016). Economic growth and carbon emissions. Economic Model..

[19] Coondoo D, Dinda S (2008). Carbon dioxide emission and income: a temporal analysis of cross-country distributional patterns. Ecol. Econ..

[20] Aller C, Ductor L, Grechyna D (2021). Robust determinants of CO2 emissions. Energy Econ..

[21] André FJ, Cardenete MA, Romero C (2009). A goal programming approach for a joint design of macroeconomic and environmental policies: A methodological proposal and an application to the Spanish economy. Environ. Manag..

[22] Sachs JD (2019). Six Transformations to achieve the Sustainable Development Goals. Nat. Sustainability.

[23] Stafford-Smith M (2017). Integration: the key to implementing the Sustainable Development Goals. Sustainability Sci..

[24] Fuso Nerini F (2018). Mapping synergies and trade-offs between energy and the Sustainable Development Goals. Nat. Energy.

[25] Campagnolo L, Davide M (2019). Can the Paris deal boost SDGs achievement? An assessment of climate mitigation co-benefits or side-effects on poverty and inequality. World Dev..

[26] Fuso Nerini F (2019). Connecting climate action with other Sustainable Development Goals. Nat. Sustainability.

[27] Von Stechow, C. et al. 2 °C and SDGs: United they stand, divided they fall? *Environmental Research Letters* **11**, (2016).

[28] Mausch K, Hall A, Hambloch C (2020). Colliding paradigms and trade-offs: agri-food systems and value chain interventions. Glob. Food Security.

[29] West PC (2019). Redesigning planning, governance, and policies to achieve multiple Sustainable Development Goals. One Earth.

[30] International Energy Agency. *Tracking Fossil Fuel Subsidies in APEC Economies*. https://www.iea.org/reports/insights-series-2017-tracking-fossil-fuel-subsidies-in-apec-economies (2017).

[31] The World Bank. The World Bank Database. https://data.worldbank.org/ (2022).

[32] Chimhowu AO, Hulme D, Munro LT (2019). The ‘New’ national development planning and global development goals: processes and partnerships. World Dev..

[33] Allen C, Metternicht G, Wiedmann T (2017). An iterative framework for national scenario modelling for the Sustainable Development Goals (SDGs). Sustain. Dev..

[34] Allen C, Metternicht G, Wiedmann T (2016). National pathways to the Sustainable Development Goals (SDGs): a comparative review of scenario modelling tools. Environ. Sci. Policy.

[35] Lindholm FA, Fossum JG, Burgess EL (1979). Application of the superposition principle to solar-cell analysis. IEEE Trans. Electron Devices.

[36] Ma WX, Fan E (2011). Linear superposition principle applying to Hirota bilinear equations. Computers Math. Appl..

[37] Dixon, P. B. & Jorgenson, D. *Handbook of computable general equilibrium modeling*. vol. 1 (Newnes, 2012).

[38] Tversky A, Kahneman D (1981). The framing of decisions and the psychology of choice. Science.

[39] Herman, J. D., Zeff, H. B., Reed, P. M. & Characklis, G. W. Beyond optimality: Multistakeholder robustness tradeoffs for regional water portfolio planning under deep uncertainty. *Water Resources Research* 7692–7713 10.1002/2014WR015338 (2014).

[40] Hurford AP (2020). Balancing services from built and natural assets via river basin trade-off analysis. Ecosyst. Serv..

[41] Hurford AP (2020). Efficient and robust hydropower system design under uncertainty - A demonstration in Nepal. Renew. Sustain. Energy Rev..

[42] Watson RT (2005). Turning science into policy: challenges and experiences from the science-policy interface. Philos. Trans.: Biol. Sci..

[43] Wellstead A, Cairney P, Oliver K (2018). Reducing ambiguity to close the science-policy gap. Policy Des. Pract..

[44] Zarkadakis, G. & Tapscott, D. Citizen assemblies. in *Cyber Republic: Reinventing Democracy in the Age of Intelligent Machines* 67–83 (MIT Press, 2020).

[45] Abbott KW (2012). Engaging the public and the private in global sustainability governance. Int. Aff..

[46] The Sustainable Development Goals Center for Africa and Sustainable Development Solutions Network. *Africa SDG Index and Dashboards Report 2020*. https://s3.amazonaws.com/sustainabledevelopment.report/2020/2020_africa_index_and_dashboards.pdf (2020).

[47] Min, Y. & Perucci, F. *Impact of COVID-19 on SDG progress: a statistical perspective*. https://www.un.org/development/desa/dpad/publication/un-desa-policy-brief-81-impact-of-covid-19-on-sdg-progress-a-statistical-perspective/ (2020).

[48] Ameli N (2021). Higher cost of finance exacerbates a climate investment trap in developing economies. Nat. Commun..

[49] International Energy Agency. *CO2 emissions from fuel combustion*. http://wds.iea.org/wds/pdf/Worldco2_Documentation.pdf (2020).

[50] Steckel JC, Hilaire J, Jakob M, Edenhofer O (2020). Coal and carbonization in sub-Saharan Africa. Nat. Clim. Change.

[51] Alova G, Trotter PA, Money A (2021). A machine-learning approach to predicting Africa’s electricity mix based on planned power plants and their chances of success. Nat. Energy.

[52] Ansari D, Holz F (2020). Between stranded assets and green transformation: fossil-fuel-producing developing countries towards 2055. World Dev..

[53] Breisinger C, Mukashov A, Raouf M, Wiebelt M (2019). Energy subsidy reform for growth and equity in Egypt: The approach matters. Energy Policy.

[54] Coady, M. D., Parry, I., Le, N.-P. & Shang, B. *Global fossil fuel subsidies remain large: an update based on country-level estimates*. https://www.imf.org/en/Publications/WP/Issues/2019/05/02/Global-Fossil-Fuel-Subsidies-Remain-Large-An-Update-Based-on-Country-Level-Estimates-46509 (2019).

[55] Arab Republic of Egypt. *Egyptian Intended Nationally Determined Contribution*. https://www4.unfccc.int/sites/ndcstaging/PublishedDocuments/EgyptFirst/EgyptianINDC.pdf (2015).

[56] The World Bank. *Understanding poverty and inequality in Egypt*. https://openknowledge.worldbank.org/bitstream/handle/10986/32812/Understanding-Poverty-and-Inequality-in-Egypt.pdf10.1596/33039 (2019).

[57] Ministry of Planning and Economic Development. *Egypt’s 2021 Voluntary National Review*. https://sustainabledevelopment.un.org/content/documents/279512021_VNR_Report_Egypt.pdf (2021).

[58] Inselberg, A. Parallel coordinates: Interactive visualisation for high dimensions. in *Trends in interactive visualization: State-of-the-art survey* (eds. Liere, R., Adriaansen, T. & Zudilova-Seinstra, E.) 49–78 (Springer London, 2009). 10.1007/978-1-84800-269-2_3.

[59] El-Katiri, L. & Fattouh, B. A brief political economy of energy subsidies in the Middle East and North Africa. *International Development Policy*10.4000/poldev.2267 (2017).

[60] Wyborn C (2019). Co-producing sustainability: reordering the governance of science, policy, and practice. Annu. Rev. Environ. Resour..

[61] Lemos MC, Morehouse BJ (2005). The co-production of science and policy in integrated climate assessments. Glob. Environ. Change.

[62] Chambers, J. M. *et al*. Six modes of co-production for sustainability. *Nature Sustainability*10.1038/s41893-021-00755-x (2021).

[63] Lakner, C., Mahler, D. G., Negre, M. & Prydz, E. B. *How much does reducing inequality matter for global poverty? World Bank Policy Research Working Paper* https://papers.ssrn.com/sol3/papers.cfm?abstract_id=3430475 (2019). 10.1007/s10888-021-09510-w PMC8890464 35261573

[64] InterAcademy Partnership working group. *Improving scientific input to global policymaking with a focus on the UN Sustainable Development Goals*. https://www.interacademies.org/publication/improving-scientific-input-global-policymaking-focus-un-sustainable-development-goals (2019).

[65] Peng W (2021). Climate policy models need to get real about people–here’s how. Nature.

[66] Faysse N (2006). Troubles on the way: an analysis of the challenges faced by multi-stakeholder platforms. Nat. Resour. Forum.

[67] Flanigan, B., Gölz, P., Gupta, A., Hennig, B. & Procaccia, A. D. Fair algorithms for selecting citizens’ assemblies. *Nature*10.1038/s41586-021-03788-6 (2021). 10.1038/s41586-021-03788-6 PMC8387237 34349266

[68] Cacuci DG, Ionescu-Bujor M (2004). A comparative review of sensitivity and uncertainty analysis of large-scale systems - II: Statistical methods. Nucl. Sci. Eng..

[69] Salem M (2020). Assessing progress towards sustainable development in the urban periphery: a case of Greater Cairo, Egypt. Int. J. Sustain. Dev. Plan..

[70] Amin-Salem, H., El-Maghrabi, M. H., Osorio Rodarte, I. & Verbeek, J. *Sustainable Development Goal Diagnostics: The Case of the Arab Republic of Egypt*. *Policy Research* https://openknowledge.worldbank.org/handle/10986/29892 (2018).

[71] Girgis, H. *et al*. *Localizing the targets of the Sustainable Development Goals on Governorate Level Study*. https://egypt.unfpa.org/en/publications/localizing-targets-sustainable-development-goals-governorate-level (2018).

[72] Banerjee, S. G., El-laithy, H., Griffin, P., Clarke, K. & Hallouda, M. Energy subsidies and the path toward sustainable reform in the Arab Republic of Egypt. in *The Quest for Subsidy Reforms in the Middle East and North Africa Region* (eds. Verme, P. & Araar, A.) 157–177 (Springer International Publishing, Cham, 2017). 10.1007/978-3-319-52926-4_6.

[73] International Monetary Fund. *Heavily Indebted Poor Countries (HIPC) initiative and Multilateral Debt Relief Initiative (MDRI)—statistical update*. https://www.imf.org/external/np/pp/eng/2016/031516.pdf (2016).

[74] Lofgren, H., Lee, R., Robinson, S., Thomas, M. & El-Said, M. *A Standard Computable General Equilibrium (CGE) Model in GAMS*. (International Food Policy Research Institute, 2002).

[75] Leontief W (1936). Quantitative input and output relations in the economic systems of the United States. Rev. Econ. Stat..

[76] Arrow KJ, Chenery HB, Minhas BS, Solow RM (1961). Capital-labor substitution and economic efficiency. Rev. Econ. Stat..

[77] Stone R (1954). Linear expenditure systems and demand analysis: an application to the pattern of British Demand. Economic J..

[78] O’Neill BC (2017). The roads ahead: Narratives for shared socioeconomic pathways describing world futures in the 21st century. Glob. Environ. Change.

[79] Fricko O (2017). The marker quantification of the Shared Socioeconomic Pathway 2: a middle-of-the-road scenario for the 21st century. Glob. Environ. Change.

[80] Riahi K (2017). The Shared Socioeconomic Pathways and their energy, land use, and greenhouse gas emissions implications: An overview. Glob. Environ. Change.

[81] World Bank. Crude oil price forecast. *Knoema* https://knoema.com/infographics/yxptpab/crude-oil-price-forecast-2021-2022-and-long-term-to-2050 (2021).

[82] Armington PS (1969). A theory of demand for products distinguished by place of production. Staff Pap..

[83] Deltares. *Development of the Eastern Nile Water Simulation Model*. (2013).

[84] Krey, V. *et al*. *MESSAGEix-GLOBIOM Documentation*. https://pure.iiasa.ac.at/id/eprint/1711510.22022/iacc/03-2021.17115 (2020).

[85] Al-Riffai, P. *et al*. *A disaggregated social accounting matrix: 2010/11 for policy analysis in Egypt*. http://ebrary.ifpri.org/cdm/ref/collection/p15738coll2/id/130736 (2016).

[86] Chepeliev M (2020). Gtap-Power data base: Version 10. J. Glob. Economic Anal..

[87] Lerch F, Ultsch A, Lötsch J (2020). Distribution optimization: an evolutionary algorithm to separate gaussian mixtures. Sci. Rep..

[88] Deb K, Jain H (2014). An evolutionary many-objective optimization algorithm using reference-point-based nondominated sorting approach, part I: solving problems with box constraints. IEEE Trans. Evolut. Comput..

[89] Zitzler E, Thiele L, Laumanns M, Fonseca CM, Da Fonseca VG (2003). Performance assessment of multiobjective optimizers: an analysis and review. IEEE Trans. Evolut. Comput..

[90] Breiman L (2001). Random Forests. Mach. Learn..

[91] Svetnik V (2003). Random Forest: a classification and regression tool for compound classification and QSAR modeling. J. Chem. Inf. Computer Sci..

[92] Hadka, D. Platypus. https://github.com/Project-Platypus/Platypus (2016).

[93] Pedregosa F (2011). Scikit-learn: machine learning in Python. J. Mach. Learn. Res..

[94] Knox S, Meier P, Yoon J, Harou JJ (2018). A python framework for multi-agent simulation of networked resource systems. Environ. Model. Softw..

[95] Bisschop, J. & Meeraus, A. On the development of a general algebraic modeling system in a strategic planning environment. in *Applications* (eds. Goffin, J.-L. & Rousseau, J.-M.) 1–29 (Springer Berlin Heidelberg, 1982). 10.1007/BFb0121223.

