# Supplementary material for: Balancing national economic policy outcomes for sustainable development
Source: Nat Commun. 2022 Aug 26;13:5041. doi: 10.1038/s41467-022-32415-9 (PMC9415247; doi:10.1038/s41467-022-32415-9)
Supplement: Supplementary file 1 — Supplementary Information [file 41467_2022_32415_MOESM1_ESM.pdf]

Supplementary Information for

Balancing national economic policy outcomes for sustainable development

Mohammed Basheer [1], Victor Nechifor [2,3], Alvaro Calzadilla [2], Claudia Ringler [4], David Hulme [5], Julien J. Harou [1,6,*]

[1] Department of Mechanical, Aerospace and Civil Engineering, The University of Manchester, Manchester, UK.

[2] Institute for Sustainable Resources, University College London, London, UK.

[3] European Commission, Joint Research Centre (JRC), Seville, Spain.

[4] International Food Policy Research Institute (IFPRI), Washington, DC, USA.

[5] Global Development Institute, The University of Manchester, Manchester, UK.

[6] Department of Civil, Environmental and Geomatic Engineering, University College London, London, UK.

*Correspondence to: julien.harou@manchester.ac.uk

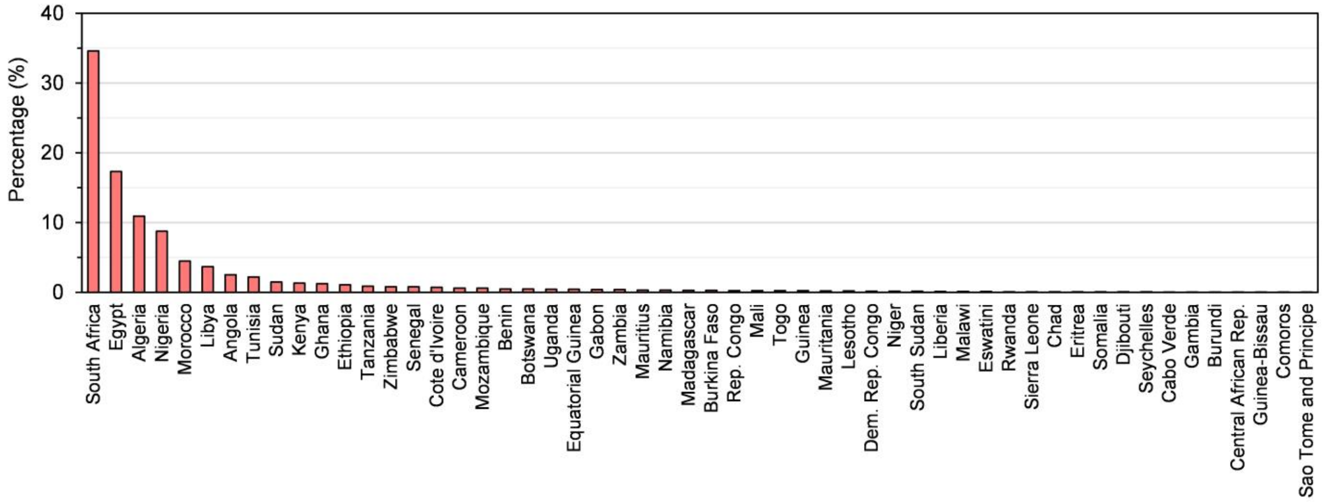

Supplementary Figure 1. Percentage contribution of African countries to total CO_2 emissions of Africa in 2016. The data depicted in this figure were acquired from the World Bank Database (https://data.worldbank.org/).

Supplementary Table 1. Details of the policy portfolios highlighted in Figures 2 and 3 in the main text. Under the income, producer, and sales tax/subsidies rows, positive and negative values indicate taxes and subsidies, respectively. Q1 to Q5 are household classes based on income quintiles from poorest to richest.

Policy instrument	Parameter	Baseline	High GDP	Low GINI	Low emissions	Compromise
**Change in total government transfers to households (%)**	Change in government transfer level compared to the baseline		-49.84	439.16	286.93	420.72
**Distribution of government transfers between household groups (%)**	Transfers to urban Q1	3.83	80.91	80.91	57.28	26.55
	Transfers to urban Q2	5.82	17.04	17.04	34.66	24.89
	Transfers to urban Q3	8.47	0.46	0.46	5.88	19.72
	Transfers to urban Q4	15.14	0.73	0.73	1.67	11.01
	Transfers to urban Q5	39.30	0.64	0.64	0.01	0.80
	Transfers to rural Q1	2.82	0.14	0.15	0.18	11.00
	Transfers to rural Q2	2.92	0.06	0.06	0.04	1.62
	Transfers to rural Q3	3.20	0.00	0.00	0.24	2.66
	Transfers to rural Q4	5.44	0.00	0.00	0.01	0.78
	Transfers to rural Q5	13.06	0.00	0.00	0.04	0.96
**Absolute values of income taxes (%)**	Urban Q1	1.49	2.32	1.58	5.54	0.64
	Urban Q2	1.45	4.45	4.45	2.06	2.14
	Urban Q3	1.43	4.43	4.20	0.91	2.55
	Urban Q4	1.40	5.07	5.07	4.18	4.30
	Urban Q5	1.40	5.56	5.69	1.11	5.32
	Rural Q1	1.49	3.16	3.16	2.18	2.90
	Rural Q2	1.47	4.69	4.69	2.63	3.32
	Rural Q3	1.44	3.81	5.71	3.80	5.53
	Rural Q4	1.46	5.86	5.86	3.11	4.88
	Rural Q5	1.42	3.36	3.36	3.98	5.66
**Absolute values of producer taxes/subsidies (%)**	Agriculture	0.00	-1.90	-1.73	1.76	2.62
	Light industry	0.00	1.50	-1.01	2.80	1.80
	Heavy industry	0.00	-0.60	-0.60	0.73	1.21
	Construction	0.00	1.72	1.72	-0.44	2.81
	Transport	0.00	-2.01	-2.81	-0.02	-2.99
	Hydroelectricity	0.00	-1.93	2.49	-0.92	-0.20
	Oil electricity	0.00	2.78	2.78	2.00	2.67
	Gas electricity	0.00	-2.57	2.60	0.28	1.53
	Solar electricity	0.00	0.66	-0.55	1.18	-0.89
	Wind electricity	0.00	2.04	2.04	1.91	0.74
	Gas processing	0.00	2.89	2.89	1.18	-0.86
	Petroleum processing	0.00	1.28	1.28	2.32	0.87
	Municipal water	0.00	1.07	1.07	-0.28	-0.16
	Public services	0.00	2.31	2.59	1.18	-2.06
	Private services	0.00	2.45	2.45	2.31	1.60
**Absolute values of sales taxes/subsidies on commodities (%)**	Agriculture	-6.78	-8.36	-8.36	-8.88	-10.15
	Light industry	2.52	4.74	4.74	4.33	3.13
	Heavy industry	3.37	6.27	6.27	2.92	4.82
	Construction	0.04	0.08	0.08	0.04	0.05
	Transport	2.82	5.15	5.15	5.34	3.37
	Gas	1.90	3.37	3.37	2.65	3.70
	Petroleum	-56.67	-30.37	-30.37	-3.04	-33.42
	Electricity	0.23	0.20	0.20	0.28	0.37
	Municipal water	0.00	0.42	0.42	-1.45	2.25
	Public services	1.57	2.26	2.26	1.02	2.43
	Private services	1.60	2.05	2.05	2.77	2.59

Supplementary Table 2. Lower and upper bounds of the different policy instruments used in the multiobjective policy search. Under the income, producer, and sales tax/subsidies rows, positive and negative values indicate taxes and subsidies, respectively. Q1 to Q5 are household classes based on income quintiles from poorest to richest.

Policy instrument	Parameter	Lower bound	Upper bound	Baseline value
**Change in total government transfers to households (%)**	Change in government transfer level compared to the baseline	0	540	
**Distribution of government transfers between household groups (%)**	Transfers to urban Q1	0	100	3.83
	Transfers to urban Q2	0	100	5.82
	Transfers to urban Q3	0	100	8.47
	Transfers to urban Q4	0	100	15.14
	Transfers to urban Q5	0	100	39.30
	Transfers to rural Q1	0	100	2.82
	Transfers to rural Q2	0	100	2.92
	Transfers to rural Q3	0	100	3.20
	Transfers to rural Q4	0	100	5.44
	Transfers to rural Q5	0	100	13.06
**Absolute values of income taxes (%)**	Urban Q1	0	6	1.49
	Urban Q2	0	6	1.45
	Urban Q3	0	6	1.43
	Urban Q4	0	6	1.40
	Urban Q5	0	6	1.40
	Rural Q1	0	6	1.49
	Rural Q2	0	6	1.47
	Rural Q3	0	6	1.44
	Rural Q4	0	6	1.46
	Rural Q5	0	6	1.42
**Absolute values of producer taxes/subsidies (%)**	Agriculture	-3	3	0
	Light industry	-3	3	0
	Heavy industry	-3	3	0
	Construction	-3	3	0
	Transport	-3	3	0
	Hydroelectricity	-3	3	0
	Oil electricity	-3	3	0
	Gas electricity	-3	3	0
	Solar electricity	-3	3	0
	Wind electricity	-3	3	0
	Gas processing	-3	3	0
	Petroleum processing	-3	3	0
	Municipal water	-3	3	0
	Public services	-3	3	0
	Private services	-3	3	0
**Absolute values of sales taxes/subsidies on commodities (%)**	Agriculture	-14	-3.5	-6.78
	Light industry	1.25	5	2.52
	Heavy industry	1.5	6.5	3.37
	Construction	0.02	0.1	0.04
	Transport	1.5	6	2.82
	Gas	1	4	1.90
	Petroleum	-80	0	-56.67
	Electricity	0.1	0.4	0.23
	Municipal water	-3	3	0
	Public services	0.75	3	1.57
	Private services	0.75	3	1.60

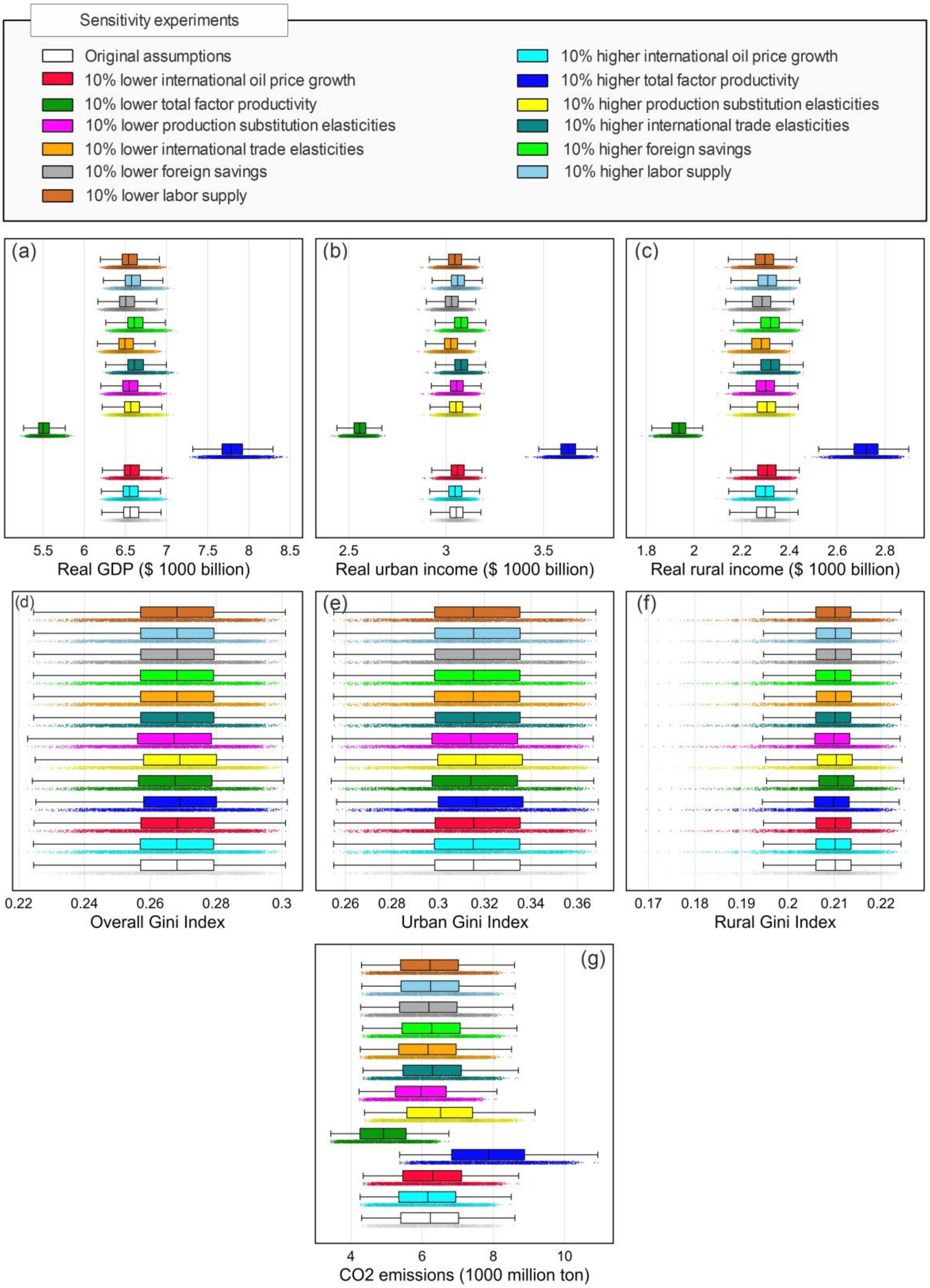

Supplementary Figure 2. Box plots of the sensitivity of the optimization objectives used in Egypt's case study application to changes in key exogenous factors of the CGE model. a Real GDP, **b** real urban income, **c** real rural income, **d** overall Gini Index, **e** urban Gini Index, **f** rural Gini Index, **g** CO_2 emissions. The ends of the boxes represent the upper and lower quartiles, the continuous vertical lines inside the boxes mark the medians, the circles show the data points, and the whiskers extend to the maximum and minimum values, excluding the outliers. The number of data points (n) in each box plot is 2494.

Supplementary Table 3. Sensitivity of policy choices of Egypt's case study application to changes in key exogenous factors of the CGE model. The fill colors of the first column correspond to the boxes with similar colors in Supplementary Figure 2.

Sensitivity experiments	Selected values and portfolios	Real GDP ($ billion)	Real urban income ($ billion)	Real rural income ($ billion)	Overall Gini	Urban Gini	Rural Gini	CO_2 emissions (million ton)
**Original**	**Highest**	7063	3195	2436	0.301	0.368	0.225	8608
	**Lowest**	6193	2873	2099	0.223	0.255	0.169	4300
	**High GDP portfolio**	7063	3024	2377	0.284	0.340	0.214	6152
	**Low Gini portfolio**	6541	3043	2328	0.264	0.311	0.207	7043
	**Low emissions portfolio**	6552	3090	2188	0.246	0.274	0.211	4633
	**Compromise**	6459	3080	2260	0.248	0.289	0.191	5862
**10% higher international oil price**	**Highest**	7054	3189	2431	0.301	0.368	0.225	8504
	**Lowest**	6187	2867	2096	0.223	0.255	0.169	4258
	**High GDP portfolio**	7054	3018	2373	0.284	0.340	0.214	6086
	**Low Gini portfolio**	6481	3166	2193	0.228	0.270	0.215	5470
	**Low emissions portfolio**	6545	3084	2184	0.246	0.274	0.211	4589
	**Compromise**	6452	3074	2255	0.248	0.289	0.191	5797
**10% lower international oil price**	**Highest**	7071	3201	2441	0.301	0.368	0.225	8713
	**Lowest**	6199	2879	2103	0.223	0.255	0.170	4343
	**High GDP portfolio**	7071	3030	2382	0.284	0.340	0.214	6219
	**Low Gini portfolio**	6495	3177	2201	0.228	0.270	0.215	5593
	**Low emissions portfolio**	6560	3095	2191	0.246	0.274	0.211	4678
	**Compromise**	6465	3086	2264	0.248	0.290	0.191	5929
**10% higher total factor productivity**	**Highest**	8465	3781	2899	0.302	0.369	0.225	10936
	**Lowest**	7285	3407	2463	0.224	0.256	0.170	5369
	**High GDP portfolio**	8465	3625	2835	0.285	0.341	0.214	7836
	**Low Gini portfolio**	7658	3748	2583	0.229	0.271	0.214	6934
	**Low emissions portfolio**	7754	3661	2578	0.246	0.275	0.211	5825
	**Compromise**	7639	3647	2662	0.248	0.291	0.191	7360
**10% lower total factor productivity**	**Highest**	5867	2685	2036	0.301	0.367	0.226	6751
	**Lowest**	5219	2410	1781	0.223	0.254	0.170	3433
	**High GDP portfolio**	5867	2510	1984	0.284	0.339	0.215	4811
	**Low Gini portfolio**	5471	2670	1859	0.228	0.270	0.215	4405
	**Low emissions portfolio**	5515	2595	1848	0.246	0.273	0.212	3670
	**Compromise**	5435	2589	1908	0.247	0.288	0.192	4660
	**Highest**	7079	3196	2436	0.302	0.369	0.226	9173

Sensitivity experiments	Selected values and portfolios	Real GDP ($ billion)	Real urban income ($ billion)	Real rural income ($ billion)	Overall Gini	Urban Gini	Rural Gini	CO_2 emissions (million ton)
**10% higher production substitution elasticities**	**Lowest**	6206	2873	2100	0.224	0.255	0.170	4382
	**High GDP portfolio**	7079	3021	2380	0.285	0.341	0.214	6378
	**Low Gini portfolio**	6501	3172	2199	0.228	0.271	0.215	5745
	**Low emissions portfolio**	6566	3090	2189	0.246	0.274	0.212	4726
	**Compromise**	6472	3081	2262	0.248	0.290	0.192	6114
**10% lower production substitution elasticities**	**Highest**	7046	3194	2435	0.300	0.367	0.225	8098
	**Lowest**	6179	2871	2098	0.223	0.254	0.169	4227
	**High GDP portfolio**	7046	3028	2376	0.284	0.339	0.214	5949
	**Low Gini portfolio**	6475	3171	2195	0.227	0.269	0.214	5338
	**Low emissions portfolio**	6539	3090	2187	0.246	0.274	0.211	4550
	**Compromise**	6445	3080	2257	0.247	0.289	0.191	5635
**10% higher international trade elasticities**	**Highest**	7130	3218	2457	0.301	0.368	0.225	8703
	**Lowest**	6238	2895	2114	0.223	0.255	0.169	4338
	**High GDP portfolio**	7130	3051	2399	0.284	0.340	0.214	6218
	**Low Gini portfolio**	6535	3194	2212	0.228	0.270	0.214	5580
	**Low emissions portfolio**	6607	3114	2205	0.246	0.274	0.211	4681
	**Compromise**	6506	3102	2276	0.248	0.289	0.191	5919
**10% lower international trade elasticities**	**Highest**	6985	3169	2411	0.301	0.368	0.225	8513
	**Lowest**	6141	2848	2081	0.223	0.255	0.169	4262
	**High GDP portfolio**	6985	2993	2352	0.284	0.340	0.214	6086
	**Low Gini portfolio**	6434	3146	2179	0.228	0.270	0.215	5481
	**Low emissions portfolio**	6490	3062	2168	0.246	0.274	0.212	4585
	**Compromise**	6404	3055	2241	0.248	0.289	0.191	5805
**10% higher foreign savings**	**Highest**	7115	3220	2454	0.301	0.368	0.225	8663
	**Lowest**	6241	2896	2115	0.223	0.255	0.169	4328
	**High GDP portfolio**	7115	3047	2395	0.284	0.340	0.214	6191
	**Low Gini portfolio**	6538	3197	2213	0.228	0.270	0.214	5565
	**Low emissions portfolio**	6602	3113	2204	0.246	0.274	0.211	4664
	**Compromise**	6509	3105	2277	0.248	0.289	0.191	5899
**10% lower foreign savings**	**Highest**	7010	3170	2417	0.301	0.368	0.225	8552
	**Lowest**	6145	2849	2083	0.223	0.255	0.169	4272
	**High GDP portfolio**	7010	3001	2360	0.284	0.340	0.214	6112
	**Low Gini portfolio**	6438	3146	2180	0.228	0.270	0.215	5496

Sensitivity experiments	Selected values and portfolios	Real GDP ($ billion)	Real urban income ($ billion)	Real rural income ($ billion)	Overall Gini	Urban Gini	Rural Gini	CO_2 emissions (million ton)
	**Low emissions portfolio**	6503	3065	2171	0.246	0.274	0.212	4602
	**Compromise**	6408	3056	2242	0.248	0.289	0.191	5825
**10% higher labor supply**	**Highest**	7085	3203	2442	0.301	0.368	0.225	8619
	**Lowest**	6211	2879	2104	0.223	0.255	0.169	4303
	**High GDP portfolio**	7085	3032	2384	0.284	0.340	0.214	6160
	**Low Gini portfolio**	6507	3179	2203	0.228	0.270	0.215	5536
	**Low emissions portfolio**	6572	3097	2194	0.246	0.274	0.212	4637
	**Compromise**	6477	3088	2265	0.248	0.289	0.191	5868
**10% lower labor supply**	**Highest**	7041	3187	2429	0.301	0.368	0.225	8596
	**Lowest**	6175	2867	2094	0.223	0.255	0.169	4297
	**High GDP portfolio**	7041	3016	2371	0.284	0.340	0.214	6144
	**Low Gini portfolio**	6469	3164	2191	0.228	0.270	0.214	5525
	Low emissions portfolio	6533	3082	2182	0.246	0.274	0.211	4629
	Compromise	6440	3073	2254	0.248	0.289	0.191	5857

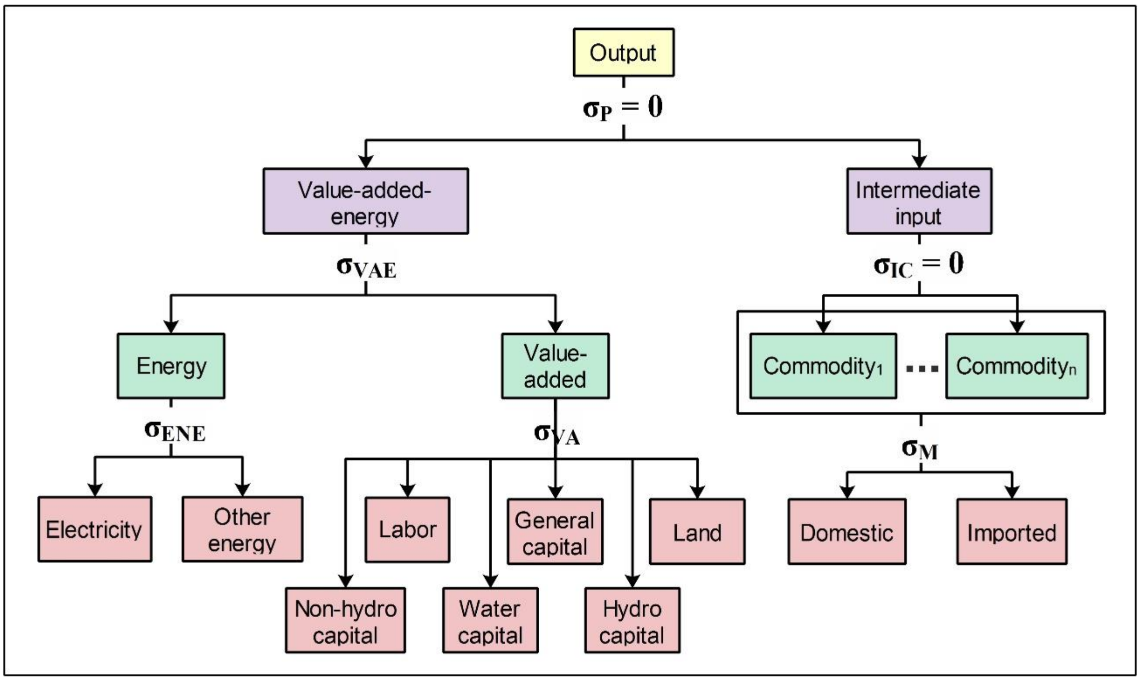

Supplementary Figure 3. Specification of production in the computable general equilibrium model.

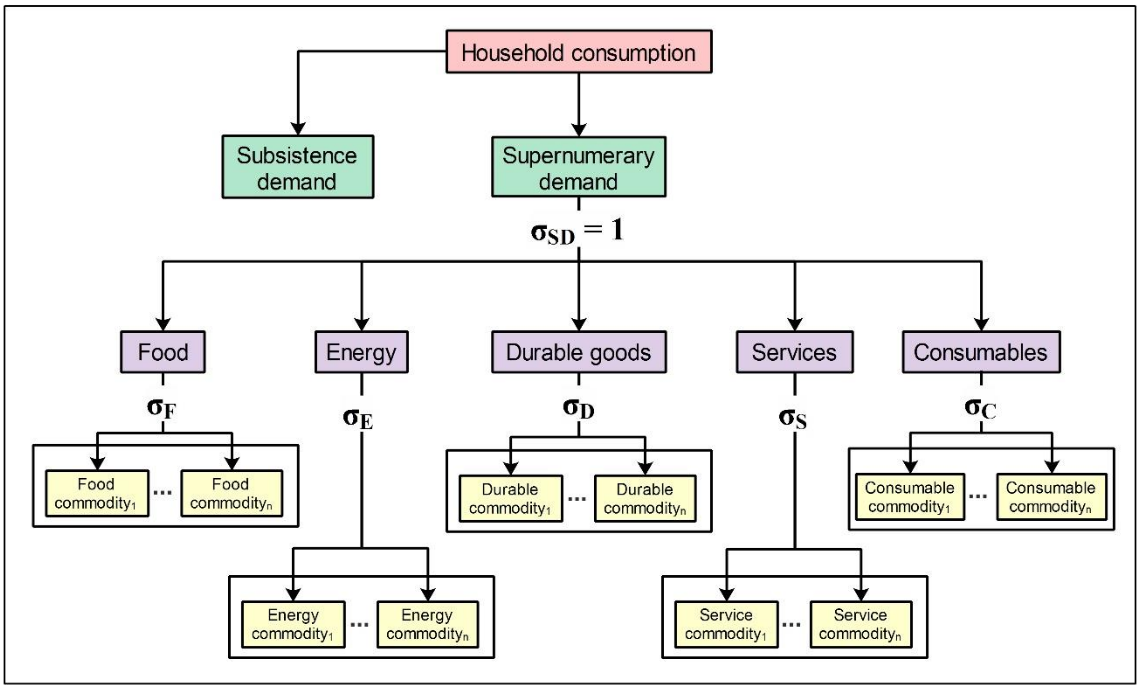

Supplementary Figure 4. Specification of household consumption in the computable general equilibrium model.

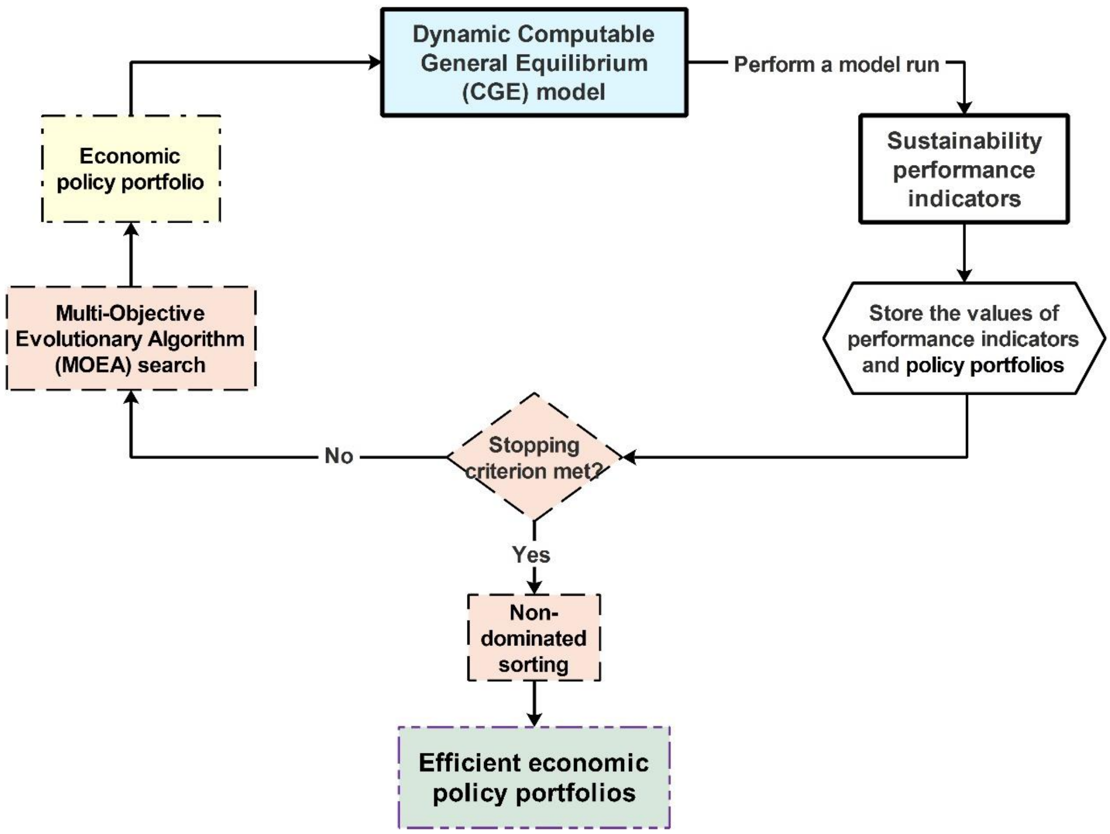

Supplementary Figure 5. Flowchart of the approach used for identifying efficient (or approximately Pareto-optimal) policy portfolios based on artificial intelligence-driven multiobjective search.

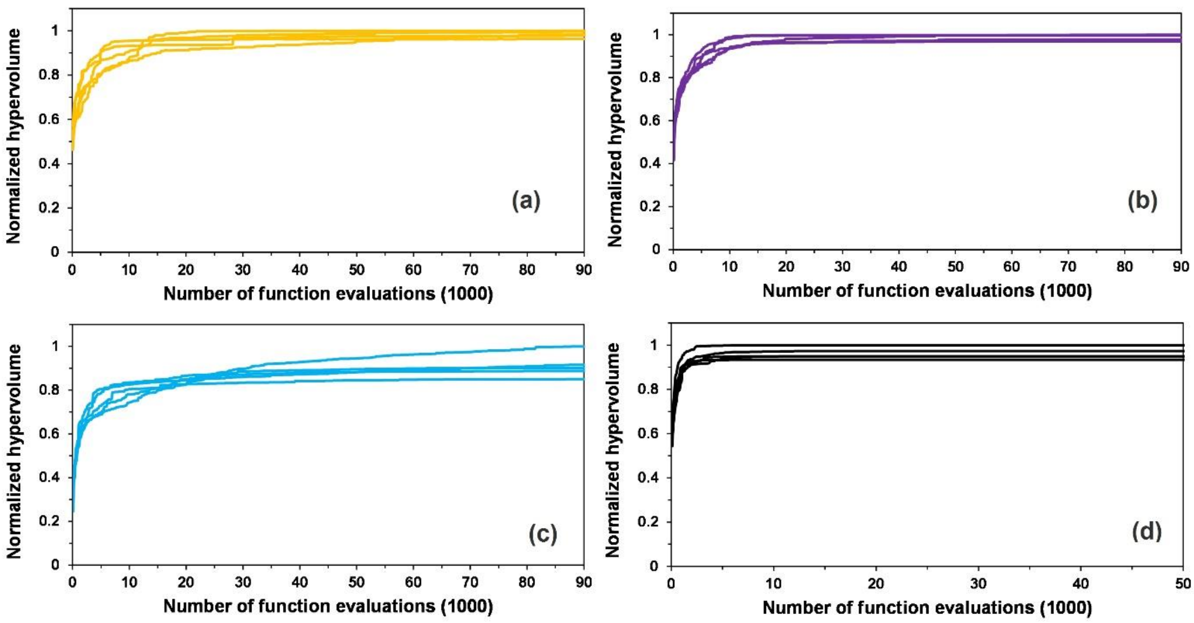

Supplementary Figure 6. Evolution of the hypervolume with five random seeds for each of the examined integrated policy strategies for the Egyptian economy: a transfer distribution and transfer level, **b** transfer distribution, transfer level, and income taxes, **c** Producer taxes/subsidies, transfer distribution, transfer level, and income taxes, and **d** Producer taxes/subsidies, sales taxes, transfer distribution, transfer level, and income taxes.

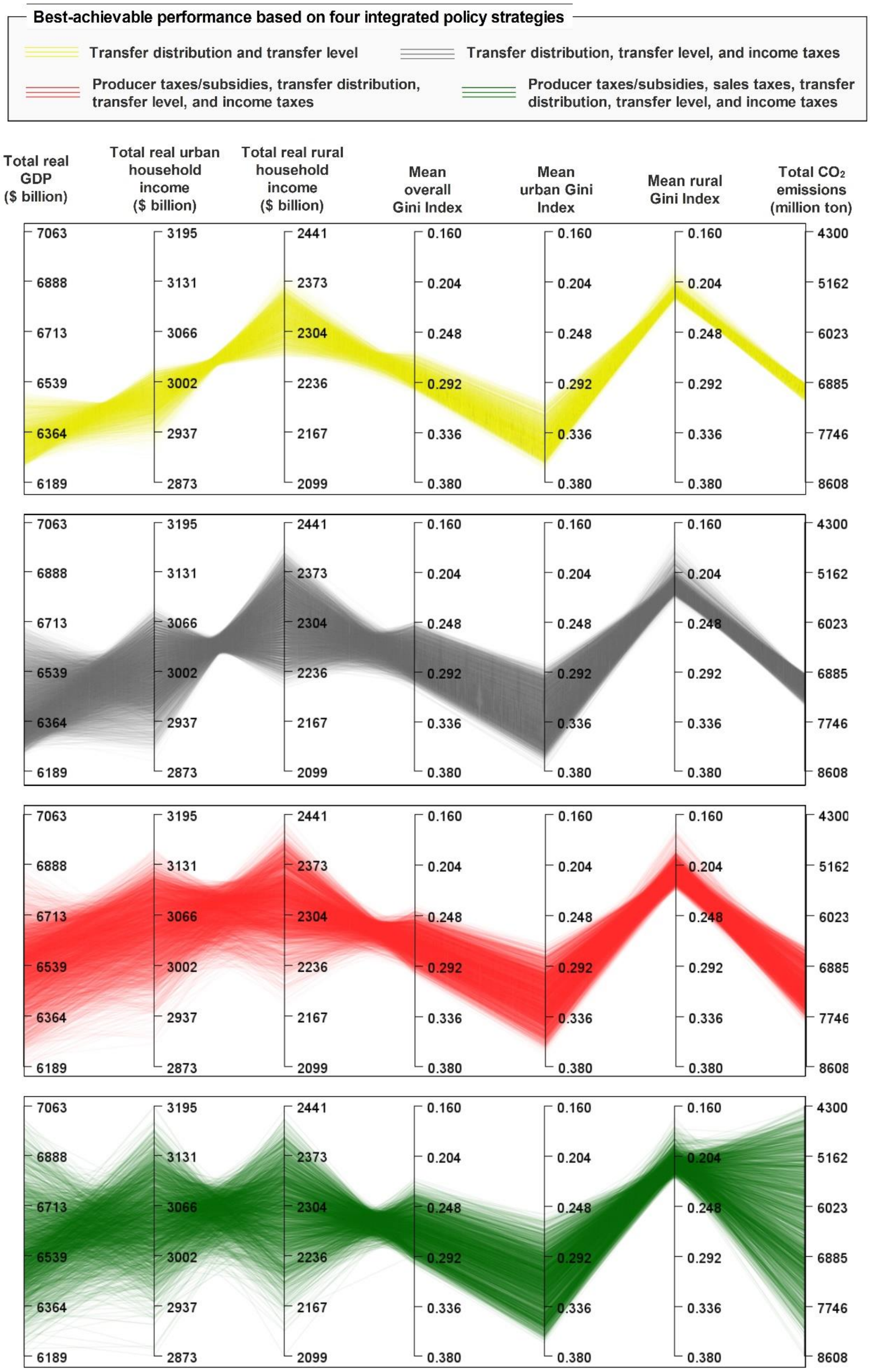

Supplementary Figure 7. Best-achievable sustainability performance of the Egyptian economy in 2021-2035 based on four integrated policy strategies.

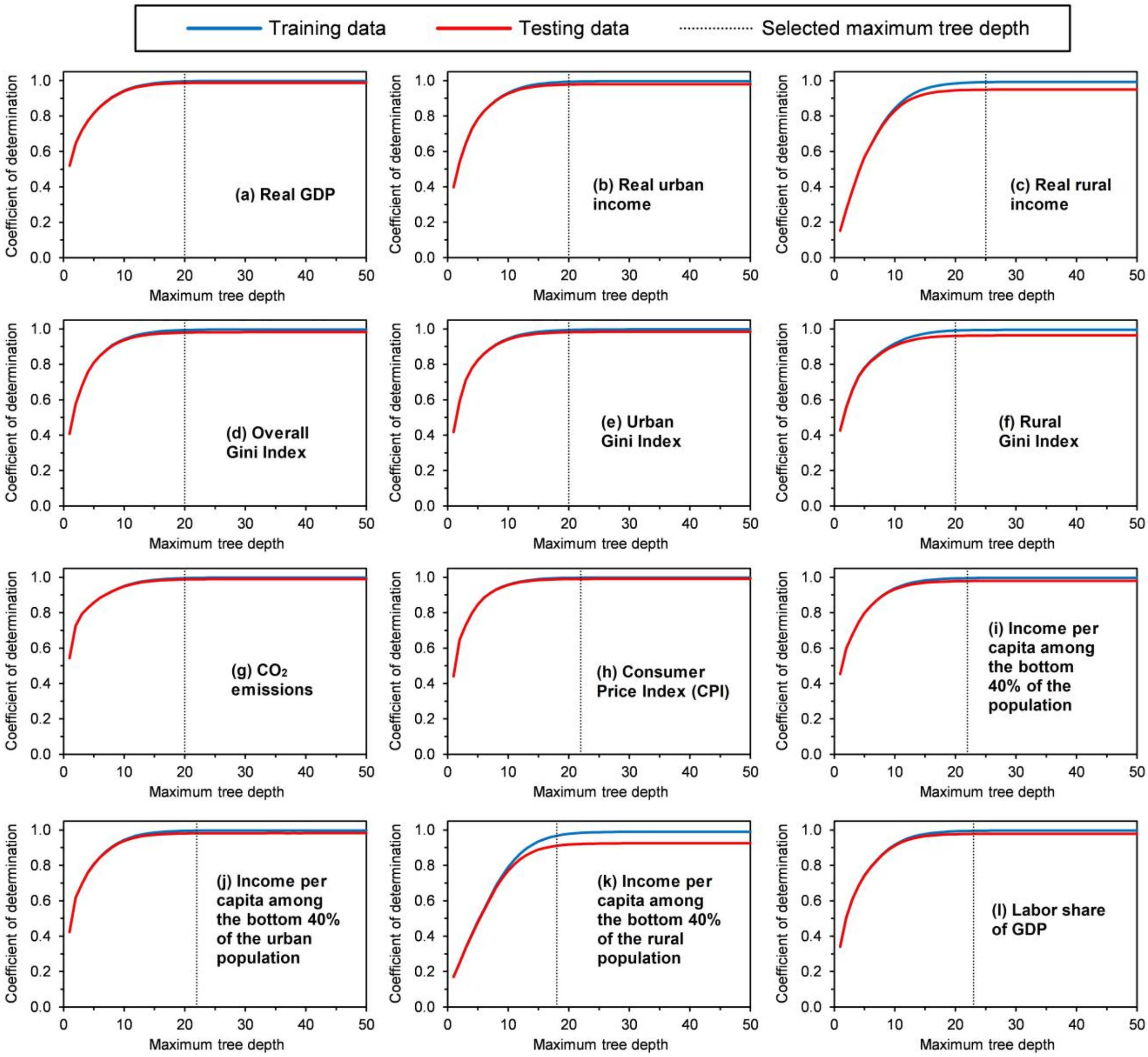

Supplementary Figure 8. Performance of the Random Forest Regression models with the training and testing data based on maximum tree depths ranging from 1 to 50. a-l performance of the machine learning models in predicting sustainability metrics. 80% of the data was used for training and 20% of the data was used for testing. A tree depth was selected for each model to avoid over- and/or under-fitting the data.

Equations of the objective function

$$RGDP = \sum_t \frac{\sum_c(\sum_h P0_c \times QH_{c,h,t} + \sum_a \sum_{c,h} PXAC0_{c,a} \times QHA_{c,a,h,t} + P0_c \times QINV_{c,t} + P0_c \times QDST_{c,t} + P0_c \times QG_{c,t} + PWE0_c \times EXR_t \times QE_{c,t} - PWM0_c \times EXR_t \times QM_{c,t})}{(1+r)^t}$$

(Supplementary Equation 1)

Where RGDP is the total discounted real GDP, $P0_c$ is the deflated market price of commodity c, $QH_{c,h}$ is the quantity of commodity c consumed by household group h, $PXAC0_{c,a}$ is the price of commodity c produced by domestic activity a, $QHA_{c,a,h}$ is the quantity of commodity c produced by the domestic activity a and consumed by household group h, $QINV_c$ is the investment demand of commodity c, $QDST_c$ is the stock change of commodity c, QG_c is the quantity of government consumption of commodity c, $PWE0_c$ is the deflated export price of commodity c, EXR is the exchange rate, QE_c is the quantity of export of commodity c, $PWM0_c$ is the deflated import price of commodity c, QM_c is the quantity of import of commodity c, r is the discount rate, and t is the simulation time step.

$$YIU = \sum_t \frac{\sum_{urb}(1 - tins_{urb,t}) \times ID_{urb,t} \times YI_{urb,t}}{(1+r)^t}$$ (Supplementary Equation 2)

Where YIU is the total discounted net real income of urban households, $tins_{urb}$ is the direct income tax rate on urban household urb, ID_{urb} is the income deflator of urban household urb, YI_{urb} is the income of urban household urb, r is the discount rate, and t is the simulation time step.

$$YIR = \sum_t \frac{\sum_{rur}(1 - tins_{rur,t}) \times ID_{rur,t} \times YI_{rur,t}}{(1+r)^t}$$ (Supplementary Equation 3)

Where YIR is the total discounted net real income of rural households, $tins_{rur}$ is the direct income tax rate on rural household rur, ID_{rur} is the income deflator of rural household rur, YI_{rur} is the income of rural household rur, r is the discount rate, and t is the simulation time step.

$$Gini = \left. \sum_t \frac{\sum_{i \in HQ} \sum_{j \in HQ} |YI_{i,t} - YI_{j,t}|}{2 \times n^2 \times \overline{YI}} \right/ T$$ (Supplementary Equation 4)

Where $Gini$ is the mean overall Gini index, HQ is a set of household groups classified by income quintiles, YI_i is the income of household group i, YI_j is the income of household group j, $\overline{YI}$ is the mean income of household groups classified by income quintiles, n is the number of household groups classified by income quintiles, t is the simulation time step, and T is the total number of simulation years.

$$GiniURB = \sum_t \frac{\sum_{i \in HQURB} \sum_{j \in HQURB} |YI_{i,t} - YI_{j,t}|}{2 \times n^2 \times \overline{YI}} \Big/ T \qquad \text{(Supplementary Equation 5)}$$

Where $GiniURB$ is the urban Gini index, HQURB are the urban household groups classified by income quintiles, YI_i is the income of urban household group i, YI_j is the income of urban household group j, $\overline{YI}$ is the mean income of urban household groups classified by income quintiles, n is the number of urban household groups classified by income quintiles, t is the simulation time step, and T is the total number of simulation years.

$$GiniRUR = \sum_t \frac{\sum_{i \in HQRUR} \sum_{j \in HQRUR} |YI_{i,t} - YI_{j,t}|}{2 \times n^2 \times \overline{YI}} \Big/ T \qquad \text{(Supplementary Equation 6)}$$

Where $GiniRUR$ is the mean rural mean Gini index, HQRUR are the rural household groups classified by income quintiles, YI_i is the income of rural household group i, YI_j is the income of rural household group j, $\overline{YI}$ is the mean income of rural household groups classified by income quintiles, n is the number of rural household groups classified by income quintiles, t is the simulation time step, and T is the total number of simulation years.

$$Emission = \sum_t \sum_{ec} QQ_{ec,t} \times CO_{ec,t} \qquad \text{(Supplementary Equation 7)}$$

Where QQ_{ec} is the quantity consumed of the CO_2 emitting commodity ec, CO_{ec} is the emission coefficient of the CO_2 emitting commodity ec, and t is the simulation time step.
