# Supplementary material for: Balancing national economic policy outcomes for sustainable development
Source: Nat Commun. 2022 Aug 26;13:5041. doi: 10.1038/s41467-022-32415-9 (PMC9415247; doi:10.1038/s41467-022-32415-9)
Supplement: Supplementary file 2 — Reporting Summary [file 41467_2022_32415_MOESM2_ESM.pdf]

nature portfolio

Corresponding author(s): Julien Harou

Last updated by author(s): May 27, 2022

Reporting Summary

Nature Portfolio wishes to improve the reproducibility of the work that we publish. This form provides structure for consistency and transparency in reporting. For further information on Nature Portfolio policies, see our Editorial Policies and the Editorial Policy Checklist.

Statistics

For all statistical analyses, confirm that the following items are present in the figure legend, table legend, main text, or Methods section.

n/a	Confirmed	
☐	☒	The exact sample size (*n*) for each experimental group/condition, given as a discrete number and unit of measurement
☒	☐	A statement on whether measurements were taken from distinct samples or whether the same sample was measured repeatedly
☒	☐	The statistical test(s) used AND whether they are one- or two-sided<br>*Only common tests should be described solely by name; describe more complex techniques in the Methods section.*
☒	☐	A description of all covariates tested
☒	☐	A description of any assumptions or corrections, such as tests of normality and adjustment for multiple comparisons
☐	☒	A full description of the statistical parameters including central tendency (e.g. means) or other basic estimates (e.g. regression coefficient) AND variation (e.g. standard deviation) or associated estimates of uncertainty (e.g. confidence intervals)
☒	☐	For null hypothesis testing, the test statistic (e.g. *F*, *t*, *r*) with confidence intervals, effect sizes, degrees of freedom and *P* value noted<br>*Give P values as exact values whenever suitable.*
☒	☐	For Bayesian analysis, information on the choice of priors and Markov chain Monte Carlo settings
☒	☐	For hierarchical and complex designs, identification of the appropriate level for tests and full reporting of outcomes
☒	☐	Estimates of effect sizes (e.g. Cohen's *d*, Pearson's *r*), indicating how they were calculated

Our web collection on statistics for biologists contains articles on many of the points above.

Software and code

Policy information about availability of computer code

Data collection	No software was used
Data analysis	The standard CGE model of the International Food Policy Research Institute (IFPRI) is open-source and freely accessible through the following link: https://www.ifpri.org/publication/standard-computable-general-equilibrium-cge-model-gams-0. The Python Network Simulation framework (Pynsim) is open-source and freely available in the following repository: https://github.com/UMWRG/pynsim. The multiobjective Non-dominated Sorting Genetic Algorithm (NSGA-III) used in the SDG economic policy design and screening framework is open-source and freely available in the following repository: https://github.com/Project-Platypus/Platypus. The Random Forest Regression, a machine learning algorithm used for understanding the drivers of sustainability performance, is open-source and freely available at: https://github.com/scikit-learn/scikit-learn.The General Algebraic Modeling System (GAMS) and the GAMS Python API can be obtained from: https://www.gams.com/. We used Python version 3.6, Pynsim version 0.1.5, Platypus version 1.0.4, and Scikit-learn version 0.24.2.

For manuscripts utilizing custom algorithms or software that are central to the research but not yet described in published literature, software must be made available to editors and reviewers. We strongly encourage code deposition in a community repository (e.g. GitHub). See the Nature Portfolio guidelines for submitting code & software for further information.

Data

Policy information about availability of data

All manuscripts must include a data availability statement. This statement should provide the following information, where applicable:

- Accession codes, unique identifiers, or web links for publicly available datasets
- A description of any restrictions on data availability
- For clinical datasets or third party data, please ensure that the statement adheres to our policy

The data that support the findings of this study are available from the corresponding author upon reasonable request at Zenodo: https://doi.org/10.5281/zenodo.6533977. The baseline population, labor, urbanization, and economic growth data of Egypt associated with the SSP-2 can be accessed from the International Institute for Applied System Analysis (IIASA) database: https://tntcat.iiasa.ac.at/SspDb/dsd?Action=htmlpage&page=10. Egypt's SAM can accessed from: http://ebrary.ifpri.org/cdm/ref/collection/p15738coll2/id/130736. Crude oil price projections can be retrieved from: https://knoema.com/infographics/yxptpab/crude-oil-price-forecast-2021-2022-and-long-term-to-2050. The GTAP-Power 10 database can be obtained from: https://www.gtap.agecon.purdue.edu/resources/res_display.asp?RecordID=5938

Human research participants

Policy information about studies involving human research participants and Sex and Gender in Research.

Reporting on sex and gender	No human participated in the experiments
Population characteristics	No human participated in the experiments
Recruitment	No human participated in the experiments
Ethics oversight	No human participated in the experiments

Note that full information on the approval of the study protocol must also be provided in the manuscript.

Field-specific reporting

Please select the one below that is the best fit for your research. If you are not sure, read the appropriate sections before making your selection.

☐ Life sciences ☒ Behavioural & social sciences ☐ Ecological, evolutionary & environmental sciences

For a reference copy of the document with all sections, see nature.com/documents/nr-reporting-summary-flat.pdf

Behavioural & social sciences study design

All studies must disclose on these points even when the disclosure is negative.

Study description	This study introduces a national-scale design framework that can enable policymakers to sift through complex, multi-sector policy spaces to identify efficient policy portfolios that balance economic, social, and environmental goals. The framework combines economy-wide sustainability simulation and artificial intelligence-driven multiobjective, multi-SDG policy search and machine learning. The framework can support multi-sector, multi-actor policy deliberation to screen efficient policy portfolios.
Research sample	In our application of the framework on Egypt, we preformed 1.8 million simulations to cover the solution space and arrive at efficient solutions.
Sampling strategy	The sampling procedure of the 1.8 million simulations (the values of the associated decision variables) was based on an iterative process between the economy-wide simulation model and a multi-objective evolutionary algorithm. Through this iterative process the multi-objective evolutionary algorithm intelligently learns and suggests new decision variables (or samples) that result in improved performance. A total of 90 thousand iterations was performed for each of the examined integrated policy strategies. The 90 thousand limit was specified to ensure convergence in the solutions provided by the multi-objective evolutionary algorithm.
Data collection	This study does not include collection and creation of primary data. All the data used are secondary data and have been obtained from the following sources. The baseline population, labor, urbanization, and economic growth data of Egypt associated with the SSP-2 can be accessed from the International Institute for Applied System Analysis (IIASA) database: https://tntcat.iiasa.ac.at/SspDb/dsd?Action=htmlpage&page=10. Egypt's SAM can accessed from: http://ebrary.ifpri.org/cdm/ref/collection/p15738coll2/id/130736. Crude oil price projections can be retrieved from: https://knoema.com/infographics/yxptpab/crude-oil-price-forecast-2021-2022-and-long-term-to-2050. The GTAP-Power 10 database can be obtained from: https://www.gtap.agecon.purdue.edu/resources/res_display.asp?RecordID=5938
Timing	This study does not include collection of primary data. All the data used are secondary data and have been obtained from the sources stated in the data availability statement.

Data exclusions	No data were excluded from the analysis.
Non-participation	No participants dropped out/declined participation
Randomization	The multi-objective evolutionary algorithm used to search for policy portfolios for Egypt was initialized using five random seeds for each integrated policy strategy.

Reporting for specific materials, systems and methods

We require information from authors about some types of materials, experimental systems and methods used in many studies. Here, indicate whether each material, system or method listed is relevant to your study. If you are not sure if a list item applies to your research, read the appropriate section before selecting a response.

Materials & experimental systems

n/a	Involved in the study
☒	☐ Antibodies
☒	☐ Eukaryotic cell lines
☒	☐ Palaeontology and archaeology
☒	☐ Animals and other organisms
☒	☐ Clinical data
☒	☐ Dual use research of concern

Methods

n/a	Involved in the study
☒	☐ ChIP-seq
☒	☐ Flow cytometry
☒	☐ MRI-based neuroimaging
